# Contrasting streamflow regimes induced by melting glaciers across the Tien Shan – Pamir – North Karakoram

**DOI:** 10.1038/s41598-018-34829-2

**Published:** 2018-11-07

**Authors:** Yi Luo, Xiaolei Wang, Shilong Piao, Lin Sun, Philippe Ciais, Yiqing Zhang, Changkun Ma, Rong Gan, Chansheng He

**Affiliations:** 1 Key Laboratory of Ecosystem Network Observation and Modeling, Institute of Geographic Sciences and Natural Resources Research, Chinese Academy of Sciences, Beijing, 100101 China; 2 University of Chinese Academy of Sciences, 19A Yuquan Rd, Shijingshan District, Beijing, 100049 China; 3 Xinjiang Institute of Ecology and Geography, Chinese Academy of Sciences, Urumqi, 830011 Xinjiang China; 4 Institute of Tibetan Plateau Research, Center for Excellence in Tibetan Earth Science, Chinese Academy of Sciences, Beijing, 100085 China; 5 Sino-French Institute for Earth System Science, College of Urban and Environmental Sciences, Peking University, Beijing, 100871 China; 6 Laboratoire des Sciences du Climat et de l’Environnement (LSCE), CEA CNRS UVSQ, 91191 Gif Sur Yvette, France; 7 College of Natural Resources and Environment, Northwest A&F University, No.3 Taicheng Road, Yangling, 712100 Shaanxi China; 8 Department of Geography, Western Michigan University, 1903 W Michigan, Ave Kalamazoo, MI 49008-5424 USA

## Abstract

The glacierized Tien Shan – Pamir – Karakoram mountain complex supplies water to about 42 million people. Yet, the knowledge about future glacial runoff in response to future climate is limited. Here, we address this issue using a hydrological model, that includes the three components of glacial runoff: ice melt, snowmelt and the runoff of rainfall over ice. The model is forced by climate projections of the CMIP5 models. We find that the three components exhibit different long-term trajectories, sometimes opposite in sign to the long-term trend in glacier impacts. For the eastern slope basins, streamflow is projected to increase by 28% (ranging from 9 to 44%, from climate model variation (CMV)) by the late 21^st^ century, under the representative concentration pathway, RCP8.5. Ice melt contributes 39% (25 to 65%, CMV) of the total streamflow increase. However, streamflow from the western slopes is projected to decrease by 5% (−24 to 16%, CMV), due to the smaller contribution of ice melt, less precipitation and higher evapotranspiration. Increasing water supply from the eastern slopes suggests more water availability for currently degraded downstream ecosystems in the Xinjiang province of China, while the likely decreasing streamflow in Central Asian rivers on the western slopes indicates new regulations will be needed.

## Introduction

**Figure 1:**
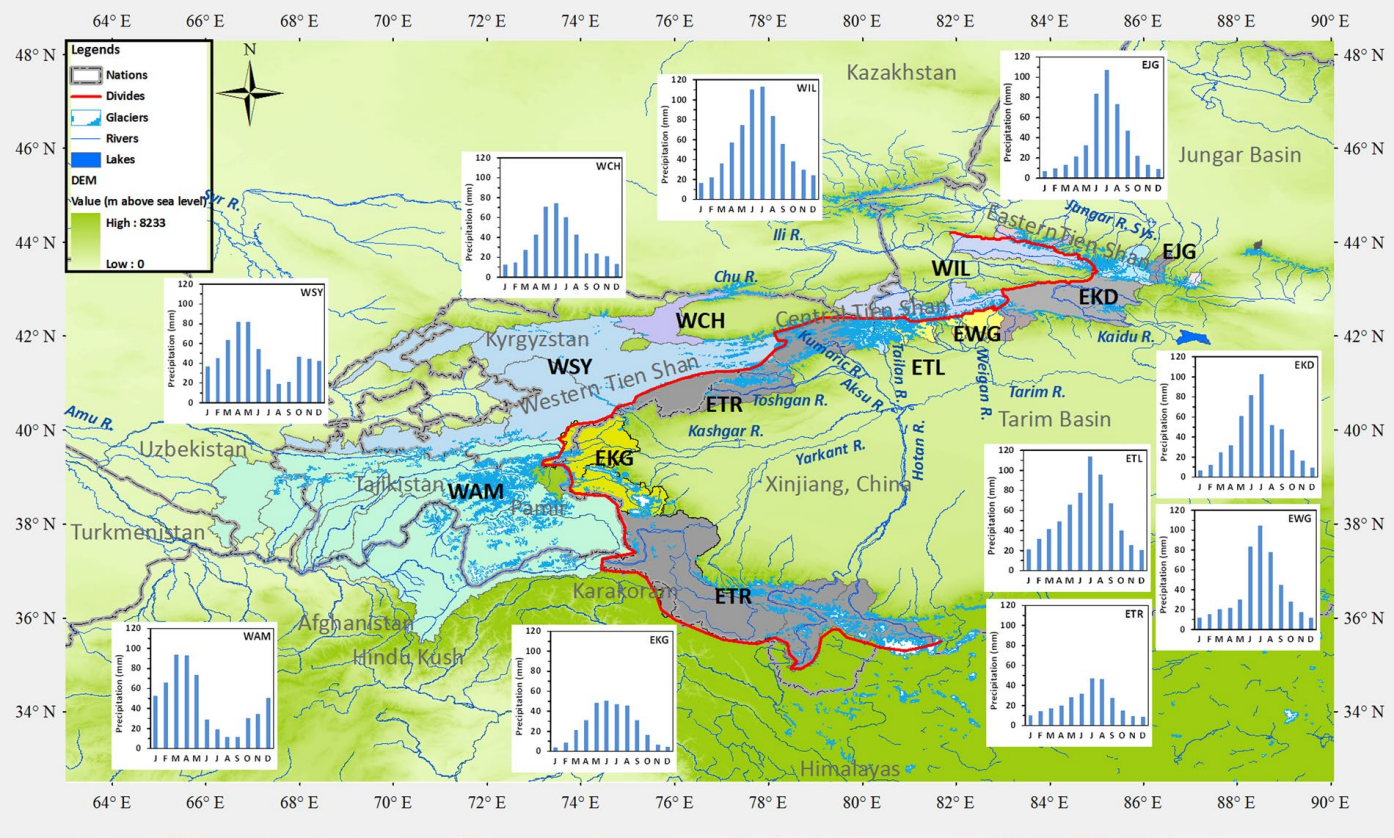
Map of the study area. Source regions are shown as coloured basins; the divide separating the eastern and western slopes along the Tien Shan – Pamir – Karakoram is shown as a red line, the international frontiers as grey double dashed lines; and the precipitation patterns as inset column graphs. The eastern slope basins (with initial letter E) are: EJG, Jungar rivers; EKD, Kaidu River; EWG, Weigan River; ETL, Tailan River; ETR, Tarim River; EKG, Kashgar River; the western slope basins (with initial letter W) are: WIL, Ili River; WCH, Chu River; WSY, Syr Darya River; and WAM, Amu Darya River.

The Tien Shan-Pamir-Karakoram (TPK) mountain complex is the “water tower” of the Central Asian countries east of the Aral Sea (Kyrgyzstan, Tajikistan, Uzbekistan, Kazakhstan, and Turkmenistan), and the Xinjiang province of western China (see Fig. 1) that was generated from the DEM data^1^ (https://earthexplorer.usgs.gov/) on ArcGIS 9 ArcMap 9.3 (http://www.esri.com). Along the Western slope of the divide, the major rivers Amu Darya (WAM), Syr Darya (WSY), and Chu River (WCH) flow to Central Asian countries, Tajikistan, Kyrgyzstan, Uzbekistan and Turkmenistan, supplying over 90% of the water resources for 22 million people in Central Asia^2^. The Ili River (WIL) originates in Xinjiang China and flows to the lake Balkhash in Kazakhstan. Along the Eastern slope of the divide, the Junagr river system (EJG) includes rivers originating from northern side of the eastern Tian Shan and flowing into the Jungar Basin; the Kaidu River (EKD) flows into the Boston Lake, and the Weigan River (EWG), Tailan River (ETL), Tarim River (ETR) and Kashgar River (EKG) flow into the Tarim Basin. The ETR consists of the Aksu River originating from the middle Tien Shan, the Yarkant River from Karakoram, and the Hotan River from the western Kunlun mountain. These rivers from the eastern TPK contributes to the water supply for 20 million people in the Xinjiang province, and sustains downstream ecosystems, which are currently degraded^3^. Changes in the magnitude or seasonality of runoff may thus have important consequences downstream to both human society and ecosystems. From inventories (see Supplementary Information), the study area is covered 27,101 glaciers with a total area of 36,539 km^2^ (see Table S1) for six main rivers on the eastern slope of the TPK mountains and four main rivers on the western slope (see Fig. 1). Glacier runoff plays an important role in regulating the streamflow and is also sensitive to climate change.

Evidence for recent climate change, given by local meteorological records over the past five decades, shows a warming rate in a range of 0.1–0.42 °C per decade^4–6^. Glacier retreat in response to this warming has been observed in the Tien Shan^7,8^ and Western and Central Pamir^9,10^, affecting water availability^11–17^. In contrast, some regions of the Karakoram, Eastern Pamir and Western Kunlun show stable glaciers or even mass gain^9,18–23^. This local response has been attributed to various causes such as the reduction of mean summer temperatures^24^ and the low sensitivity of snowfall to warming in winter^25^.

As pointed out by Sorg *et al*.^4^ and Unger-Shayesteh *et al*.^5^, there is a lack of integrated assessment of the impacts of climate change on the future evolution of the TPK glaciers and their consequences for river discharge. One difficulty in assessing river discharge in this region is the unknown future behavior of glacial runoff, which is considered in this study as the sum of ice melt, snowmelt and the runoff of rainfall over ice^26^. These three processes have distinctly different responses to climate change. Ice melt has a long-term response to climate change, while the supraglacial snowmelt and rainfall runoff over ice have much faster changes than it. Because ice melt is difficult to measure directly for a larger area^26^, we used an glacier - enhanced SWAT (Soil Water and Assessment Tool) model^27^ to simulate it. This model (see Supplementary Information) describes glaciers as hydrological response units (GHRUs) and calculates ice melt based on glacier mass balance and an area-volume scaling relationship^28,29^. A GHRU is divided into elevation bands of equal intervals, and the mass balance of a glacier is the sum of the balance of each band. It also calculates the supraglacial snowmelt and the runoff of rainfall over ice components of glacial runoff. The GHRU approach has been incorporated into the distributed Soil Water Assessment Tool (SWAT)^30,31^ to simulate catchment hydrological changes. We calibrated and validated the combined glacier-hydrology model using daily (in Xinjiang basins) and monthly (in Central Asian basins) streamflow records from 55 gauging stations (see Fig. Sm2) and observed glacier area change. We tested (see Supplementary Information) the ability of the model to reproduce observed streamflow changes from 1961 to 2007 and basin-scale glacier changes between the 2000s and the 1960s, forced by precipitation from APHRODITE (the Asian Precipitation – Highly Resolved Observational Data Integration Towards Evaluation of Water Resources Data Integration^32^) and temperature data from PGMFD (the Princeton Global Meteorological Forcing Dataset^33^). For the simulated and observed monthly streamflow of those ten rivers, the Nash-Sutcliffe efficiency criterion^34^ varies between 0.33 and 0.94 with a mean of 0.79 and standard deviation (SD) of 0.11; the relative bias ranges between −30 and 23% with a mean of 2% and SD of 9%; the coefficient of determination of a linear regression between observed and modelled discharge over the gauged period 1961–2007 ranges from 0.77–0.94, with a mean of 0.87 and SD of 0.06 in the ten studied rivers. Linear regression between the simulated and observed glacier area change has a correlation coefficient of 0.97 with a coefficient of determination of 0.91 and zero intercept for the ten basins considered (see Figs Sm4, Sm5 and Sm6 in Supplementary Information: modelling and data). These results suggest that the glacio-hydrological model parameterizations are sufficiently realistic to allow use of the model to investigate future changes. Hydrological changes were projected to 2100 using CMIP5 climate models (a total of 16 models involved) outputs under the Representative Concentration Pathways (RCPs) 2.6, 4.5 and 8.5 climate scenarios. The word “pathway” was nice shorthand coined by the developers of the RCP concept, meaning time series of anthropogenic greenhouse gas concentration. The different RCP represents different pathway that may lead to a target radiation forcing till 2100^35^. Four combinations (dry-and-cold, dry-and-warm, wet-and-cold, and wet-and-warm) were derived for each RCP at the 10^th^ and 90^th^ percentiles of the projected temperature and precipitation changes, and the model runs closest to the percentiles were eventually selected^36^ (see Fig. Sm8 and Table Sm2 in Supplementary Information). The outputs of the climate models were downscaled to 0.25° × 0.25° and bias corrected by the ‘delta change’ approach^14,37–39^ to generate daily temperature and precipitation series to drive our model for simulating past and future hydrological changes. Ensemble means of the four combinations in 2016–2045 and 2066–2095 were used to represent changes in the future.

## Results

### Historical changes

The retrospective analysis of the period 1966–1995 indicates a recent mean glacial-runoff (incl. ice melt runoff and supraglacial components consisting of snow melt and runoff from rainfall over ice)^26^ contribution to streamflow in the range of 4 to 61%, in which ice melt alone contributed from 1 to 22% for the ten major rivers. As part of the glacial runoff, the catchment ice-melt contributions varied between 30 and 55% with a mean of 48% over the TPK river basins. The modelled variations between rivers are principally due to differences in upstream glacier coverage and in climate (see Table Sm1). On the eastern slope EWG, ETL, EKG, and ETR river basins, which are heavily glacierized, the glacial-runoff contribution to annual streamflow ranges between 40 and 61%, while the ice melt contributes 19 to 22%. Glaciers in this region are usually located in high elevation headwater sub-basins so that the glacial-runoff and ice-melt contribution ratios decrease as the river progresses downstream^14^. For example, in the WAM on the western slope, the glaciers are concentrated in the Pyanj and Vakhsh tributary sub-basins, where the glacier-runoff contribution to the annual streamflow of the whole basin represents less than 13%, and the ice-melt contribution 7%. The fractions of meltwater runoff and rainfall-runoff in streamflow for the ten rivers in Fig. 1 are shown in Fig. S1 and Table S2.

### Future changes in glacier area and volume

The model estimated that in the future both glacier area and ice-water storage will decline significantly, yet with regional differences. Relative to the 1960s under the low-warming scenario RCP2.6, between 36 and 55% (mean 52%) of the glacier area is projected to disappear by 2045 in the western slope basins, compared to 19 to 59% (mean 26%) loss for the eastern slope. Among all the scenarios, RCP2.6 projects the smallest increase in temperature; however, the TPK glacier area will continue to decrease in the twenty-first century, especially in its second half, indicating that glacier retreat will be a general trend under atmospheric warming in the future. Under the high and continuous warming scenario of RCP8.5, the glacier-area loss is similar to RCP2.6 by 2045, given the small differences in temperature and precipitation at that time between both scenarios^35^. However by 2095 under RCP8.5 coupled with continued global increases in greenhouse gases and temperatures, the mean glacier-area loss will become larger than in RCP2.6, with a 70 to 81% (mean 79%) loss on the western slope, and 52 to 93% (mean 59%) on the eastern slope. Meanwhile, in the RCP8.5 climate scenario, by 2095, relative to the 1960s, the loss of ice-water storage ranges between 46 and 94% (mean 54%) on the eastern slope and 64 to 68% (mean 65%) on the western slope; in two river basins, EKD in the eastern slope and WCH in the western slope, most of the glaciers will have disappeared. Glaciers decrease less on the eastern slope because they experience a larger increase in precipitation, and because their average size is greater, they are less vulnerable to warming. Details of projected glacier changes are presented in Fig. S2 and Table S3.

### Future changes in glacier runoff and its components

**Figure 2:**
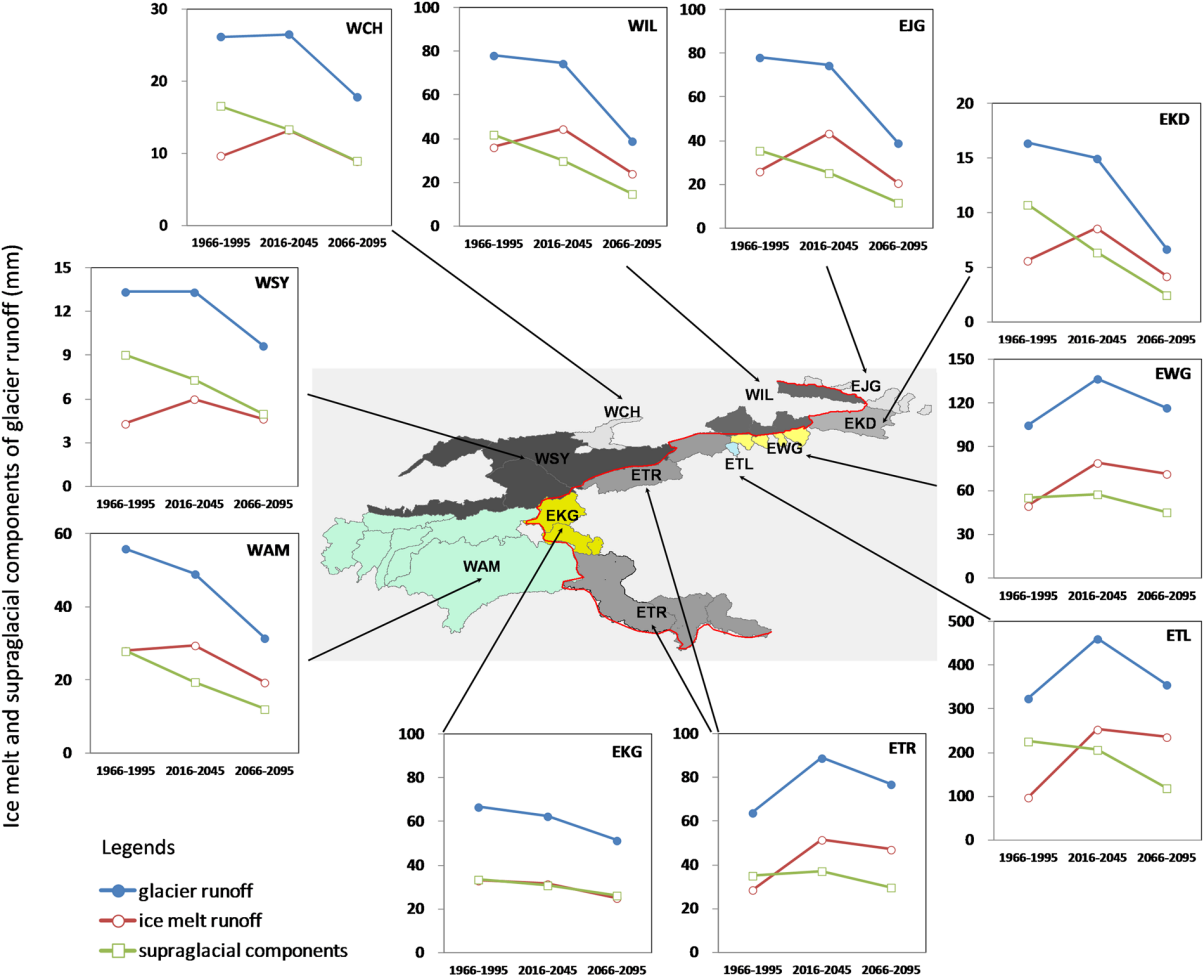
Long-term change patterns in total glacial runoff and its components for the main rivers in the Tien Shan-Pamir-Karakoram for RCP4.5 (see Fig. S2 for RCP2.6 and RCP8.5). See Fig. 1 for labelling of river catchments.

Long-term trajetories of glacier runoff components, ice melt runoff and supraglacial components (snow melt and rainfall runoff on ice) were compared (see Figs 2 and S3). Ice melt is simulated to reach a maximum in the middle of the twenty-first century under both RCP2.6 and RCP4.5. Under RCP8.5, ice melt is projected to increase continuously to 2100 for the heavily glacierized basins EWG, WTL, and ETR. In contrast, total glacial runoff is found to decline until the late twenty-first century in all the ten rivers except for the ETL, EWG and ETR. In the most heavily glacierized eastern slope basins, the EWG, ETL, and ETR (see Fig. 1), changes in ice melt dominate changes in glacial runoff. In other less glacierized western slope basins, declining supraglacial snowmelt explains most of the decreasing trend of total glacial runoff. In WIL, WSY, and WAM in the western slope, and the less glacierized EJG and EKD in the eastern slope, in the first half of the twenty-first century, supraglacial snowmelt dominates the glacial runoff trend, even where ice melt and the supraglacial component have opposite trends. During the late twenty-first century, ice melt drops below its historical level in most of the basins except for the initially heavily glacierized EWG, ETL, and ETR (see Figs 2 and S3). Most recently, Huss and Hock projected the global glacier runoff changes until 2100^17^. They indicated that in the glacier fed Aral Sea (Amu and Syr) and Tarim rivers, annual glacier runoff is projected to rise until roughly the middle of the century, followed by steadily declining glacier runoff thereafter under RCP 4.5. For the Tarim River, the projected long-term patterns are compatible. However, for the WAM and WSY, we projected that the turning points have already passed (see Fig. 2). The difference might be due to the different climate and glacier runoff models that were used in our studies, which imply that the uncertainties caused by climate and glacier runoff models are significant issues to be addressed.

### Future changes in streamflow

**Figure 3:**
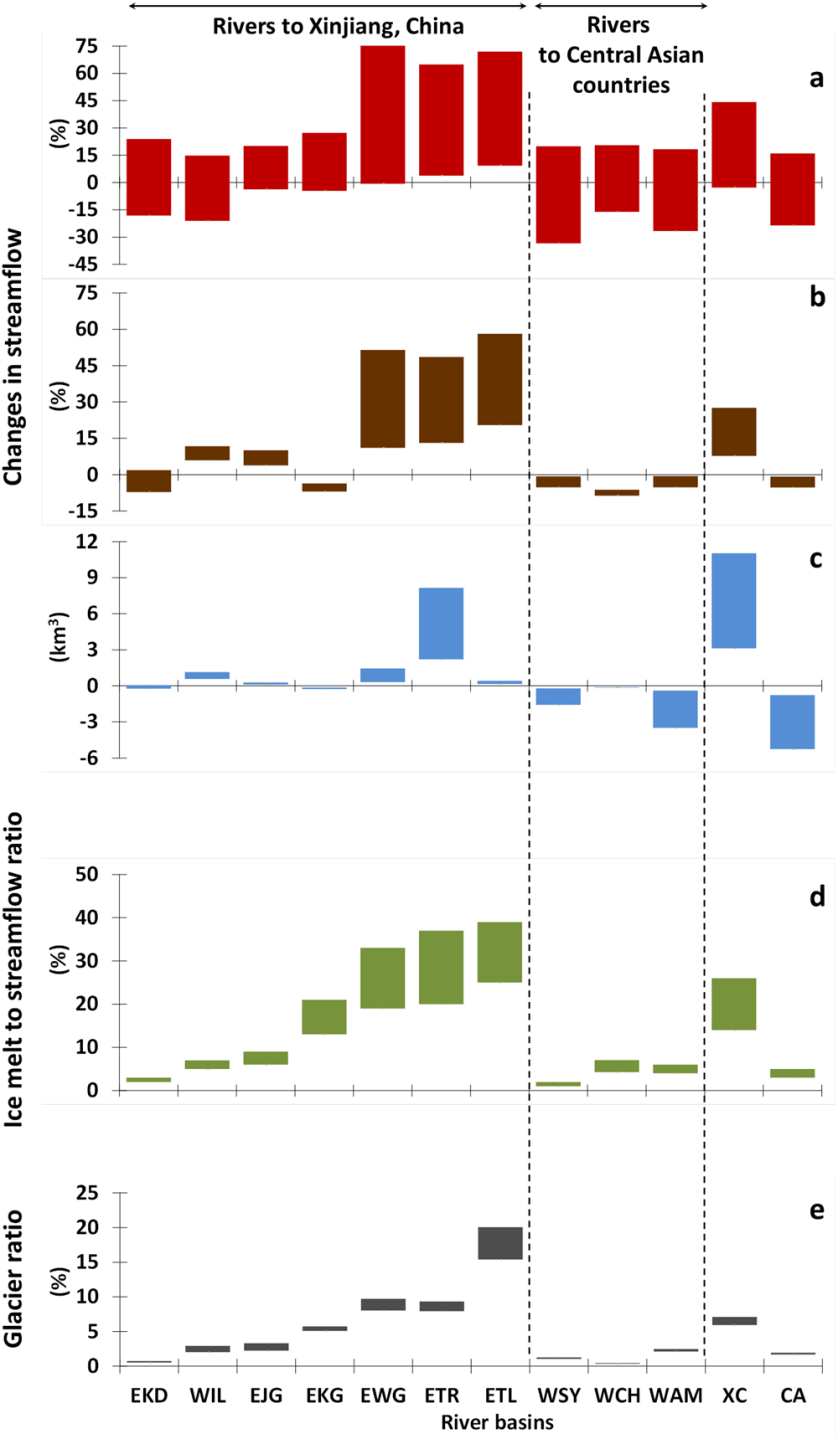
Projected change in streamflow relative to 1966–1995 for the main rivers in the Tien Shan-Pamir-Karakoram to 2100 under climate change scenarios RCP2.6, RCP4.5, and RCP8.5. (**a**) The range in streamflow change by multiple climate models under multiple climate scenarios in percentage. (**b**) The range in ensemble mean streamflow change under multiple climate scenarios in percentage, and (**c**), in flow volume. (**d**) Ensemble means of ice melt to streamflow ratio under multiple climate scenarios in 2066–2095. (**e**) Ensemble means of glacier area coverage ratio under multiple climate scenarios in 2066–2095. See Fig. 1 for labelling of catchments; rivers delivering water to Xinjiang, China are labelled XC; and rivers to Central Asian countries, CA.

Future changes in streamflow were presented in Fig. 3a,b by percentage and Fig. 3c by volume for the major rivers, respectively. In Fig. 3, the major rivers were categorized into two groups, one into Xingjiang China (XC) and another into Central Asian (CA) countries, repsectively. In Fig. 3a, the variations cover the uncertainties of the climate models (CMV) and scenarios. In Fig. 3b,c, the variations cover the uncertainties of climate change scenarios.

As a result of changes in glacial runoff and changes in precipitation in the non-glacierized parts of each catchment, streamflow is projected to increase in eastern slope rivers (and in WIL), but to decrease in western slope rivers between 1966–1995 and 2066–2095. For RCP2.6, streamflow increases in eastern slope catchments by 8% (−3 to 20%, climate model variation, CMV) between 1966–1995 and 2066–2095; for RCP8.5, streamflow also increases by 28% (9 to 44%, CMV), with ice melt contributing 39% (25 to 65%, CMV) of this signal. Taking into account the range of future uncertainties resulting from climate models and scenario differences, the streamflow in the Xinjiang rivers is *very likely*^40^ (94% probability) to increase in 2066–2095 (see Figs 3 and S4). For the western slope rivers WAM, WSY, and WCH delivering water to Central Asian countries, the streamflow is set to decrease by an average of 1% (−9 to 7%, CMV) under RCP2.6 and by 5% (−24 to 16%, CMV) under RCP8.5 between 1966–1995 and 2066–2095, respectively (see Fig. 3). The streamflow in the Central Asian rivers is *more likely*^40^ to decrease (60% probability) than increase, when all the uncertainties of climate models and scenario differences are considered.

Under RCPs 2.6, 4.5, 8.6 and relative to 1965–1995, the streamflow is expected to change by 6.5–8.3 billion cubic meters (bcm) for Xinjiang rivers and (−)0.6–1.2 bcm for Central Asian rivers in 2045–2066, respectively. In 2066–2095, the change is expected to be 3.1–11.0 bcm for Xinjiang rivers and (−)5.3 – (−)0.8 bcm for Central Asian rivers, respectively (see Fig. 3c).

### Attribution of streamflow changes

By comparing long-term changes in the water balance components, i.e., precipitation, change in ice storage, and evapotranspiration (see Supplementary Information), we found that increasing ice melt and precipitation are the dominant reasons for streamflow increase in rivers on the eastern slope. In the heavily glacierized basins of the EWG, ETL, and ETR, where today’s glacier coverage is above 5% and ice melt makes up over 13% of the streamflow, the future increase in streamflow is dominated by loss of ice-water storage (see Figs 3 and S4). In the less glacierized basins of the EKD, the streamflow increase is attributed to higher precipitation. In the moderately glacier-covered EJG basins, both ice melt and precipitation support the increased streamflow. On the other hand, on the western slope, ice melt contributes only marginally to streamflow (see Figs 3d and S4) and increasing evapotranspiration and decreasing precipitation are the main causes of streamflow decline in the WCH, WSY and WAM river catchments (see Fig. S4).

Figure 3e describes the projected glacier ratios in 2095 under RCPs 2.6, 4.5 and 8.5. It is also found that the ice melt to streamflow ratios (see Fig. 3d) and changes in streamflow (see Fig. 3a–c) are closely related to the glacier ratios (see Fig. 3e) in the major rivers. For higher glacier ratio, the varation ranges is wider, which indicates more sensitive to warming temperature. Meanwhile, the higher the glacier ratio is, the more the ice melt will contribute to the streamflow, and the more likely streamflow will increase.

### Tipping points

**Figure 4:**
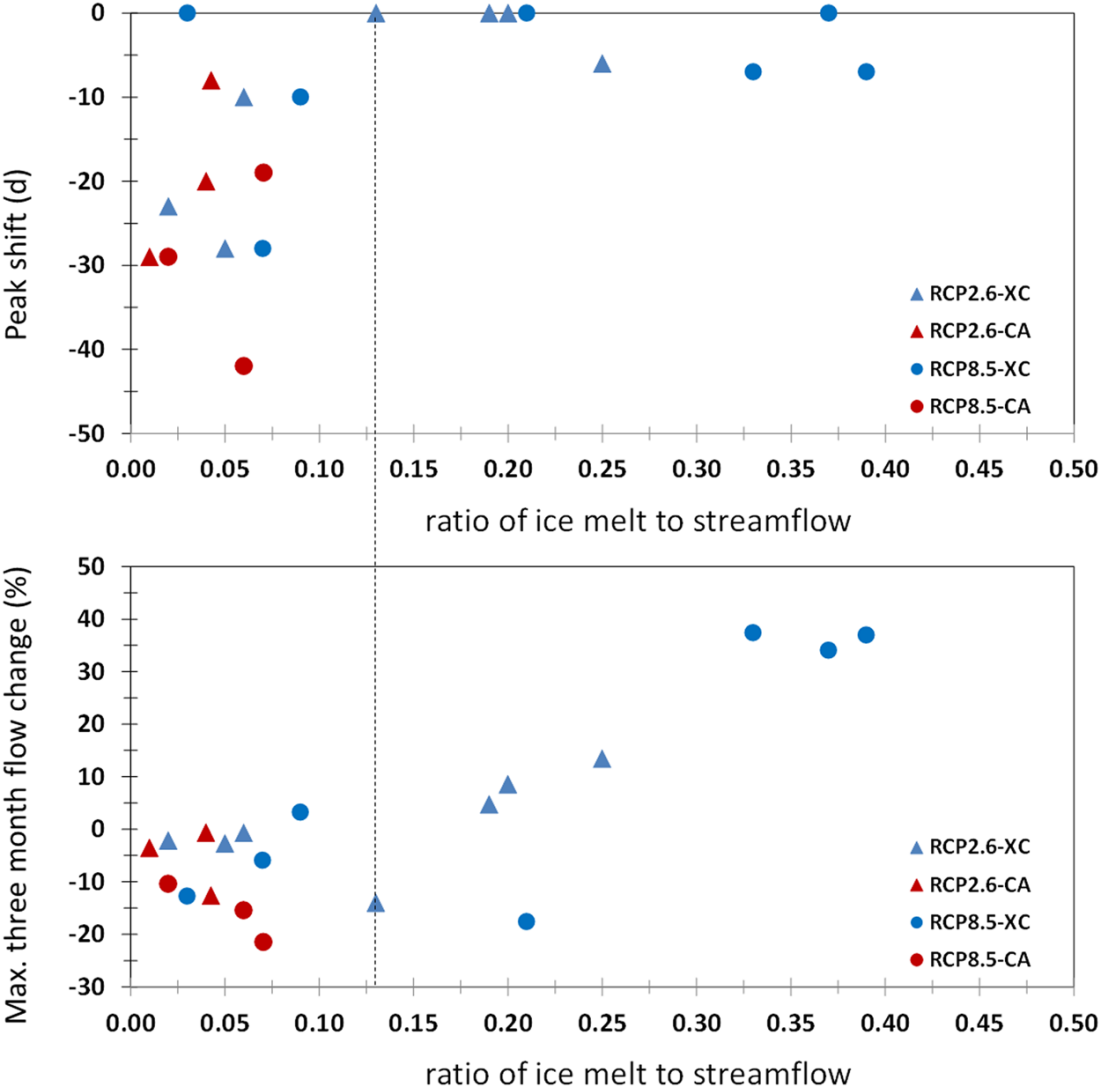
Shift towards earlier peak in seasonal river flow (upper panel) and changes in the amplitude of maximum flow (average of the three months with highest values, lower panel). Values are plotted against the ratio of annual ice melt to streamflow averaged over 2066–2095, between historical (1966–1995) and future (2066–2095) conditions for the TPK rivers. XC, for rivers delivering water to Xinjiang, China; CA: for rivers to Central Asian countries.

Changes in hydrographs due to snowmelt, ice melt, and streamflow are more apparent for rivers on western slopes than on eastern ones (see Fig. S5). The snowmelt seasonal contribution to hydrographs shows a change in peak timing and values, but no obvious shift in the ice melt seasonal contribution is seen under the warming climate (see Fig. S5). With steadily increasing temperature during the twenty-first century, snowmelt seasonal hydrographs shift earlier for all the 10 rivers studied; by ~10 days in 2016–2045 and 11 to 32 days in 2066–2095 depending on the RCP considered, relative to 1966–1995. No obvious seasonal shift of streamflow hydrographs is projected for eastern slope rivers but a shift towards earlier maximum streamflow on the western slope is found, by 18 days in 2016–2045 and by 42 days in 2066–2095 under RCP8.5 (see Fig. 4). High-flow volume increases were noted over three months (June–August) for highly glacierized basins on the eastern slope. However, in the western slope rivers, a reduction can be seen, by 15 to 21% under RCP8.5 in 2066–2095. Ice melt stabilizes the peak flow timing on the one hand, but causes an increasing high-flow volume on the other when ice-melt contribution is more than 13%. However, when the ice-melt contribution is below 13%, there is a detectible advance in peak timing and a negative change in maximum streamflow values (see Fig. 4).

## Discussion and Conclusion

In the contiguous region of the TPK, ranging from the south slope of Karakoram to Himalaya, runoff in the twenty-first century is expected to increase consistently due to increasing glacier melt and precipitation until the 2050s^14,36^. In some of the TPK tributary catchments, the streamflow is projected to increase until the 2020 s but then begins to decline until the end of the twenty-first century^11,15^. It is worth noting that this study projects similar long-term trend patterns of melt water and streamflow in those tributary catchments, but with later turning points and slower decline in glacier area, melt, and streamflow up to the end of the twenty-first century. This projected behavior is possibly due to uncertainties caused by the glacial melt modelling approach and the GCM scenarios.

As most of the similar studies in the high mountain regions, scarcity of the ground-based climatic data is always an important issue to deal with. Different proxy data base may be used to drive the simulation. Uncertainty caused by the precipitation data remains an unaddressed issue. Andermann *et al*. (2011) evaluated quite a few data products in the Hymalays waersheds and indicated that APHRODITE data, a product processed from gauge stations, which gives the best precipitation estimates when compared to independent ground observations^41^. They also indicated that the lack of stations at high elevation limits the accuracy of this data set; more ground-based observations could allow for better conclusions drawn from any atmospheric-related studies in the Hymalas region; this might be also correct for the Tien Shan – Pamir – Karakoram Range.

Evapotranspiration is another important water balance component which might be a source of uncertainties. It depends upon inputs of the historical and future climate, and the theoretical approaches (see Supplementary Information). However, evapotranspiration is perhaps a grayer zone than other water balance items in the alpine hydrology due to data scarcity in data for input and for output evaluation of the theoretical approaches.

In summary, glacier mass loss in response to climate change will lead to contrasting water supply situations for the downstream users of TPK water resources. The increase in water supply on the eastern slope will favour water users downstream in Xinjiang during the next decades, albeit with a significant loss of ice. This extra water may provide an opportunity to restore the degraded riparian forest ecosystems, e.g., the *Populus Euphratica* forests growing along the lower reach of the Tarim River^3^. However, the fact that streamflow will not increase, or will more likely decrease, in the WCH, WSY and WAM rivers indicates that human use of water should not exceed current levels if we are to avoid risks of trans-boundary water disputes between Central Asian countries. Earlier shifts in the streamflow hydrographs and reduction of the high-flow volume in the WSY and WAM rivers also implies that water regulations should be improved in Central Asian countries, to allow hydropower generation in upper-reach countries (Kyrgyzstan and Tajikistan) and summer irrigation in the lower-reach countries (Uzbekistan, Kazkhakstan, and Turkumenistan)^2^.

## Methods

We used a fully distributed glacio-hydrological model, applying a GHRU approach^27^ to simulate glacier processes and dynamics. The model is suitable for the analysis of large river basins. The GHRU approach explicitly simulates glacier mass balance and transient area–volume dynamics interactively on a time-step of one day. Each GHRU is divided into 100 m elevation bands, to account for spatial variation in precipitation and temperature over the altitudinal range. Glacier mass balance is simulated for each elevation band. The mass-balance components included are: the accumulation of snow, transformation of snow to ice, sublimation/evaporation, melting and refreezing. In the model, supraglacial snowmelt is calculated first, and then ice melt if the snow disappears leaving ice exposed to air temperature. The model neglects the effects of debris-cover found on some glaciers. Both snowmelt and ice melt were calculated using degree-day factors. Rain falling on to ice is assumed to run off directly as “runoff of rainfall over ice”. A volume-area scaling relationship was used to simulate the transient change of glacier area and volume, updated by ice mass changes. In contrast to other glacio-hydrological models that lump glaciers and glacier patches in an individual grid square into a single virtual glacier^42,43^, the GHRU approach simulates individual glaciers and therefore can capture the different transient climate-change responses of glaciers of different sizes. Glacial runoff was separated into ice melt, supraglacial snowmelt, and runoff of rainfall over ice.

The GHRU approach was incorporated into the SWAT^30,31^ hydrological model to simulate basin-wide glacio-hydrological processes. The glacier-enhanced SWAT model simulates glacial and hydrological processes and routes water through the channel systems to the outlet.

Glacier-area data were compiled from the World Glacier Inventory (WGI) data set released by the National Snow and Ice Data Center (NSIDC), the Tien Shan and Pamir glacier inventories of Aizen^44^, and the first (1960s) and second (2010s) Chinese glacier inventories^8^. A total of 27,101 glaciers were modelled, for details of each basin refer to Table S1.

The forcing meteorological data came from meteorological stations, daily precipitation data for 1951‒2007 (with a spatial resolution of 0.25° × 0.25°) from APHRODITE^32^, and daily maximum and minimum temperatures for 1948–2008 (with 0.5° × 0.5° spatial resolution) from PGMFD^33^. Post-processing of these data sets is described in the Supplementary Information.

The glacier-enhanced SWAT model was calibrated and validated using daily or monthly streamflow records from 55 gauging stations and the basin-wide glacier-area change in the Manas, Kaidu, Weigan, Tailan, Kashgar and Tarim basins on the eastern slope, and the Ili, Chu, Syr and Amu basins on the western slope of the TPK mountain complex (see Fig. 1 and Table S1), which shows that the model reproduces the observed streamflow and basin-scale glacier changes reliably.

We projected the hydrological changes to 2100 using CMIP5 climate model outputs under the Representative Concentration Pathways (RCPs)^35^ 2.6, 4.5 and 8.5. Within the CMIP5 model space, we constructed four composite contrasting climate change trajectories for each of the five sub-regions of the TPK (see Table S1) by taking the 10th and 90th percentiles of their projected precipitation and temperature changes between 1966–1995 and 2066–2095, so as to account for model uncertainties. The selected four GCMs represent wet-and-cold, wet-and-warm, dry-and-warm, dry-and-cold states of the model space^14^. The GCM outputs were downscaled using the ‘delta change’ approach^14,37–39^ to generate a time series of daily data^14^ and to project future changes separately. We used an ensemble mean of the simulation forced by the four GCMs to derive the long-term change trends of hydrological regimes and water availability.

Annual catchment water balances included the input precipitation, the outputs of evapotranspiration and streamflow at the outlet, and the storage changes in ice, soil moisture, and groundwater. The simulation results indicated that the annual storage changes of soil moisture and groundwater over the long term are negligible. Thus the storage-change terms reduce to ice change only.

The *ET*_*a*_ is derived from the potential evapotranspiration (*PET*) for all land use and land cover types in this study. The SWAT model provides several approaches for estimating *PET*, and we used the Hargreaves (H-G) method^31^. The H-G method is temperature based, i.e., it needs only the daily maximum, minimum, and mean temperatures as input. Meanwhile, it takes into account of effect of solar radiation on *PET* by inclusion of the extraterrestrial radiation. The extraterrestrial radiation depends only upon solar declination, geographic latitude, and the angular velocity of earth’s rotation. There is not any parameter in the H-G method that needs calibration and validation based on historical data series, while the historically based parameter might not be suitable for future climate as discussed in literatures^45–47^. Application of the H-G method is also found in similar studies in Karakoram – Himalayan range watersheds^14,42,48,49^.

Comparing changes of the water balance components averaged over 2016–2045 and 2066–2095 to that over 1966–1995, we identified the patterns of hydrological variables and the reasons for streamflow changes over the long term. Further details of the methods used are given in the Supplementary Information.

## Electronic supplementary material

 Supplementary Information
