# Supplementary material for: Contrasting streamflow regimes induced by melting glaciers across the Tien Shan – Pamir – North Karakoram
Source: Sci Rep. 2018 Nov 7;8:16470. doi: 10.1038/s41598-018-34829-2 (PMC6220244; doi:10.1038/s41598-018-34829-2)
Supplement: Supplementary file 1 — Supplementary Information [file 41598_2018_34829_MOESM1_ESM.pdf]

Contrasting streamflow regimes induced by melting glaciers across the Tien Shan – Pamir – North Karakoram

Yi Luo[1, 2, 3*], Xiaolei Wang[1, 2], Shilong Piao[4, 5*], Lin Sun[1], Philippe Ciais[6], Yiqing Zhang[2], Changkun Ma[7], Rong Gan[2], Chansheng He[8]

1. Institute of Geographic Science and Natural Resources Research, Chinese Academy of Sciences, Beijing 100101, China

2. University of Chinese Academy of Sciences 19A Yuquan Rd, Shijingshan District, Beijing, 100049, China

3. Xinjiang Institute of Ecology and Geography, Chinese Academy of Sciences, Urumqi, 830011, Xinjiang, China

4. Institute of Tibetan Plateau Research, Center for Excellence in Tibetan Earth Science, Chinese Academy of Sciences, Beijing 100085, China.

5. Sino-French Institute for Earth System Science, College of Urban and Environmental Sciences, Peking University, Beijing 100871, China

6. Laboratoire des Sciences du Climat et de l'Environnement (LSCE), CEA CNRS UVSQ, 91191 Gif Sur Yvette, France.

7. College of Natural Resources and Environment, Northwest A&F University, No.3 Taicheng Road, Yangling 712100, Shaanxi, China

8. Department of Geography, Western Michigan University, 1903 W Michigan Ave Kalamazoo MI 49008-5424, USA

* Corresponding to: Yi Luo, luoyi@igsnrr.ac.cn; Shilong Piao, slpiao@pku.edu.cn

Supplementary Information-modelling and data

Table of Contents

Contrasting streamflow regimes induced by melting glaciers across the Tien Shan – Pamir – North Karakoram 1

Supplementary Information-modelling and data 2

1 Large-scale glacio-hydrological model 5

1.1 Glacio-hydrological modelling 5

1.1.1 Glacier Hydrological Response Unit 5

1.1.2 Glacier mass balance 6

1.1.3 Glacier volume-area scaling relationship 6

1.1.4 Glacier melt and melt components 8

1.2 The catchment glacio-hydrological model 8

1.3 Catchment water balance 9

2 Study area 10

3 Description of data 11

4 Description of climate and streamflow characteristics of the river basins 15

5 Glacio-hydrological model calibration and validation 17

6 Climate change and hydrological response simulation 23

6.1 Selection of GCM runs 23

6.2 Downscaling and hydrological simulation 26

6.3 Projected temperature and precipitation change in the future 27

7 References 30

List of Figures

Fig. Sm1: description of the glacier hydrological response unit (GHRU) approach. Abbreviations: Q_{gs}, supraglacial snowmelt runoff; Q_{gi}, glacier ice-melt runoff; Q_{ir}, rainfall-runoff on ice surface; Q_g, glacial runoff; H_{max} and H_{min}, top and bottom elevations of a glacier, respectively. The background picture is a clip from Google Earth (Image©2015 DigitalGlobe, https://www.google.com/earth/) 5

Fig. Sm2: a map depicting the locations of discharge gauges (green dots), meteorological stations (red dots) and glaciers (light blue patches) in the study area. On the eastern slope, the basins are: EJG, Jungar rivers; EKD,

Kaidu River; EWG, Weigan River; ETL, Tailan River; ETR, Tarim River; EKG, Kashgar River; on the western slope, basins are: WIL, Ili River; WCH, Chu River; WSY, Syr River; and WAM, Amu River. The map was generated from the DEM data[22] (https://earthexplorer.usgs.gov/) on ArcGIS 9 ArcMap 9.3 (http://www.esri.com). 11

Fig. Sm3: monthly mean temperature and precipitation over 1966–1995 in source regions of the main rivers in western and eastern slopes of the Tien Shan – Pamir – Karakoram, respectively. See Fig. Sm2 for labelling of river catchments....................16

Fig. Sm4: the *NSE* (top) and *PBias* (bottom) evaluation indices for comparison of the simulated and observed monthly discharge at gauging stations in the source regions of the main rivers in the Tien Shan-Pamir-Karakoram(blue column for calibration and brown column for validation) 20

Fig. Sm5: regression analysis for the simulated and observed discharge at gauging stations in the main river basins in the Tien Shan-Pamir-Karakoram. A total of 54 gauges were used in this study. For clarity, the gauges are grouped on the basis of the main rivers or main tributaries in the study area. See Fig. Sm2 for labelling of river catchments....................21

Fig. Sm6: comparisons of simulated and observed streamflow (with error bars showing the standard deviation) for the ten main rivers in the Tien Shan – Pamir – Karakoram. The monthly streamflow values are averaged over the period 1961-1989 for the eastern slope rivers, EJG (Jungar river system), EKD (Kaidu River), EWG (Weigan River), ETL (Tailan Rvier), ETR (Tarim Rvier), EKG (Kashgar River), and the western slope river, WIL (Ili River); the other western slope rivers, WCH (Chu River), WSY (Syr River), and WAM (Amu River) are averaged over the period 1961-1985 22

Fig. Sm7: comparison of simulated glacier area change to observed change in benchmark catchments in the Tien Shan – Pamir – Karakoram 23

Fig. Sm8: an example of GCM run selection: climate change projection for the source region of Amu River under RCP4.5. Abbreviations: dP, relative difference between mean precipitation in 2066–2095 and 1966–1995; dT, absolute difference between mean temperatures in 2066–2095 and 1966–1995. Circles indicate GCM runs; solid boxes the 10% and 90% percentiles; and the crosses indicate the selected GCMs runs....................24

Fig. Sm9: projected changes in temperature (top) and precipitation (bottom) relative to 1966–1995 in the upstream river basins in the Tien Shan-Pamir-Karakoram. See Fig. Sm2 for labelling of river catchments....................28

List of tables

Table Sm1: seasonal distribution of precipitation and streamflow of the major rivers in the Tien Shan-Pamir-Karakoram, averaged over 1966–1995. See Fig. Sm2 for labelling of river catchments....................16

Table Sm2: main sensitive parameters calibrated in the glacier-enhanced SWAT model for the 24 catchments in the Tien Shan mountains (Reference range values are adopted from literatures[4,34,42,44,45]) 19
Table Sm3: lists selected GCM runs for climate projection in source regions of the main rivers in the Tien Shan-Pamir-Karakoram...24
Table Sm4: projections of the temperature and precipitation changes in source regions of the main rivers in the Tien Shan-Pamir-Karakoram. See Fig. Sm2 for labelling of river catchments...28

1 *Large-scale glacio-hydrological model*

1.1 Glacio-hydrological modelling

1.1.1 Glacier Hydrological Response Unit

We used a glacier hydrological response unit (GHRU) approach to simulate glacier changes. We consider each glacier in a sub-basin to be a GHRU as described in Fig. Sm1. Each GHRU is divided into elevation bands of equal intervals, accounting for spatial heterogeneities in precipitation and temperature over an elevation profile. Within each elevation band, we assume that precipitation and temperature are uniform. Glacier area in each elevation band is initially determined through the glacier area-elevation distribution curve, which changes with time. For each elevation band, glacier mass balance simulation is calculated on a daily basis. Glacier mass balance is the sum of each of the elevation bands. Glacier area is updated with the new glacier mass via the volume-area scaling relationship. As input, the GHRU module needs top and bottom elevations and glacier area-elevation distribution profile to initialize a simulation. These data are available in an inventory database or are retrieved from glacier-cover maps and DEM.

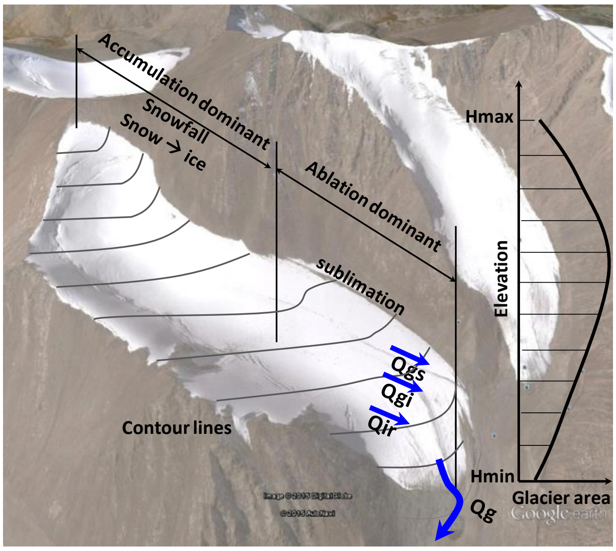

Fig. Sm1: description of the glacier hydrological response unit (GHRU) approach. Abbreviations: Q_{gs}, supraglacial snowmelt runoff; Q_{gi}, glacier ice-melt runoff; Q_{ir}, rainfall-runoff on ice surface; Q_g, glacial runoff; H_{max} and H_{min}, top and bottom elevations of a glacier, respectively. The background picture is a clip from Google Earth (Image©2015 DigitalGlobe, https://www.google.com/earth/)

1.1.2 Glacier mass balance

The mass balance includes two main components: (1) glacier mass accumulation and (2) glacier ablation. The glacier mass balance is given as

$$\frac{dW_g}{dt} = -(1-f)M - Sl + F \qquad \text{(S1)}$$

where W_g is the depth of water equivalent of ice in mm, M is the melt rate of ice in mm day^{-1}, f is the fraction of meltwater that refreezes, SL is the sublimation rate of glacier in mm day^{-1}, F is glacier accumulation rate in mm day^{-1}, and t is the time-step in days. Based on observations of the Qiyi Glacier in the Qilian Mountains in northwest China, a value of 0.2 is set for f[1].

Supraglacial snowmelt is calculated with a temperature-index approach[2], where the degree day factor (DDF) varies sinusoidally between a minimum value at the winter solstice and a maximum value at the summer solstice, reflecting the annual cycle of incoming solar radiation[3]. Ice melt is simulated using the same approach as for snowmelt but with a different DDF. Calculation of other mass balance components is described in detail by Luo *et al.*[4].

1.1.3 Glacier volume-area scaling relationship

Clarke *et al.* (2015)[5] presented a template for projecting regional glacier changes with the dynamic and mass balance equations. As an alternative, the glacier volume-area scaling relationship proposed empirically by Chen and Ohmura (1990)[6] has been widely applied to estimate regional or even global glacier changes under climate change and their impacts on water availability and sea level rise[7-12]. This relationship is often incorporated into hydrological models to simulate glacio-hydrological processes at catchment or regional scales[4,13-15]. The scaling relation is generally expressed as:

$$V = cS^{\gamma} \qquad \text{(S2)}$$

where V in km^3 and S in km^2 are the volume and surface area of a glacier, respectively; c and γ are scaling factors. Bahr *et al.* (2015)[16] reviewed the volume-area scaling relationship, indicating that the scaling relationship is physically sound; it can be derived theoretically through dimensional analysis or the stretching symmetry transformation from the constitutive, motion, and continuity equations of glaciers. Huss and Farinotti compared the scaling relations to their Δh parameterization approach and found that the former caused higher uncertainties. They indicated that a single scaling relation might not be valid for the entire global; and the higher uncertainty might be explained by the unsuitability of conventional scaling relations for nonsteady state and/or markedly dendritic glaciers[17]. However, Bahr et al. (2015) emphasized that the derivative of the scaling relationship assumes only that the ice flow follows the Glen's flow law. No other assumptions such as steady and/or equilibrium states are required. Considering the difficulties in obtaining the basal topography of the individual glaciers, the numerical models are not inherently more accurate than the scaling relationship. It should be noticed that the volume-area scaling relation is suitable for a population of glaciers, which are in either steady or non-steady states[16]. Its scaling exponent γ is theoretically equal to 1.375 for valley glaciers, while the scaling factor c may change both spatially and temporally, although many previous studies have taken it as a constant[16]. Liu *et al.* (2003) [18] obtained a value for γ of 1.35 and a value for c of 0.04 based on 691 glaciers in the Qilian Mountains which are close to the study area. We used these two scaling constants in this study.

Glacier volume is connected to glacier area and depth of water equivalent of glacier mass by:

$$V = 10^{-6} \times \frac{W_g \cdot S \cdot \rho_w}{\rho_i} \quad \text{(S3)}$$

where ρ_w is the bulk density of water, usually taken as 1000 kg m^{-3}; ρ_i is the bulk density of ice, usually taken as 900 kg m^{-3}.

1.1.4 Glacier melt and melt components

With the GHRU approach, snow melts before ice for glacier-ice covered by snow. This approach assumes that a fraction of the meltwater (supraglacial snowmelt or ice-melt) is refrozen while the other fraction leaves as runoff. With precipitation on the ice surface as rain, it is assumed that the rainfall leaves the ice surface as runoff, i.e., "the runoff of rainfall over ice". The sum of the three components is then the glacier melt runoff, expressed as[19]:

$$Q_{sr} + Q_{ir} + Q_{rr} = Q_{gr} \qquad \text{(S4)}$$

where Q_{sr} is supraglacial snowmelt runoff; Q_{ir} is glacier ice-melt runoff; Q_{rr} is rainfall-runoff on the ice surface; and Q_{gr} is glacier melt runoff. Equation (S4) is used to analyze the reasons for changes in glacier melt and to isolate the effects of glaciers on streamflow due to ice-melt.

1.2 The catchment glacio-hydrological model

SWAT (Soil Water Assessment Tool, http://swat.tamu.edu/) is a physically-based distributed catchment hydrological model[20]. It is widely used for a broad range of climatic, soil, land-cover, and management conditions. For spatial heterogeneities in land surface hydrology, SWAT delineates the catchment into sub-basins. For spatial heterogeneities within sub-basins, it adopts the hydrological response unit (HRU) approach. This approach lumps together land areas with unique land-cover, soil and management combinations in the sub-basin. Hydrological processes (e.g., plant growth, energy balance, soil evaporation and plant transpiration, water body evaporation, snow pack and snowmelt, runoff generation and infiltration, etc.) are simulated at the HRU phase and the water yield routed via tributary and main channels to the basin outlet. The SWAT model documents[21] are accessible at http://swat.tamu.edu/documentation/. The officially released SWAT model does not simulate glacier hydrology.

SWAT is an open source model, and users are free to add new modules to meet specific simulation needs.

We added the GHRU module to SWAT to meet the simulation needs of catchment-scale glacio-hydrological processes. For catchment or regional scale application, the data for glacier module parameterization and initialization are readily available in the inventories and/or glacier coverage maps from remote sensing images. Unlike the lumped HRU approach of SWAT, we take each individual glacier as a GHRU.

1.3 Catchment water balance

The water balance of a glacier-free catchment differs from that of a glaciated catchment: the latter has an additional water source from ice-melt. The water balance for the catchment comprises: the input term of precipitation (P), the output terms of evapotranspiration (ET_a) over the catchment and discharge (Q) at the outlet, and then the changes in water storage (ΔS) during the water balance period, expressed as:

$$P + \Delta S - ET_a = Q \quad \text{(S5)}$$

The change in water storage includes change in glacier-water storage and that in other storage terms such as soil moisture, aquifers and surface water bodies, expressed as:

$$P + \Delta S_i + \sum_{l=1}^{n} \Delta S_l - ET_a = Q \quad \text{(S6)}$$

where i is glacier ice; and l is soil moisture, and aquifers.

The simulation results of this study indicated that changes in soil water and aquifer storage over the periods 1966–1995, 2016–2045 and 2066–2095 are negligible, equation (S6) is thus reduced to:

$$P + \Delta S_i - ET_a = Q \quad \text{(S7)}$$

Glacier ice-water storage change is calculated as:

$$\Delta S_i = W_g(t_0) - W_g(t_1) \quad \text{(S8)}$$

where t_0 and t_1 are the start time and end time of the water balance analysis.

$$\Delta Q = \Delta P + \Delta(\Delta S_i) - \Delta ET_a \quad \text{(S9)}$$

where ΔQ, ΔP, $\Delta(\Delta S_i)$, and ΔET_a are increments of these items in 2016–2045 or 2066–2095 relative to 1966–1995. Equation (S9) is used to identify the main components causing streamflow change.

Equations (S7) – (S9) provide another set of tools for analyzing the long-term change in streamflow under climate change in glaciated watersheds.

It should be mentioned that ET_a is derived from the potential evapotranspiration (*PET*) for all land use and land cover types in this study. The SWAT model provides quite a few approaches for estimating *PET*, and we used the Hargreaves (H-G) method [21]. The H-G method is temperature based, i.e., it needs only the daily maximum, minimum, and mean temperatures as input. Meanwhile, it takes into account of effect of solar radiation on *PET* by inclusion of the extraterrestrial radiation. The extraterrestrial radiation depends only upon solar declination, geographic latitude, and the angular velocity of earth's rotation.

2 Study area

We studied ten main rivers in the Tien Shan, Pamir, and Karakoram ranges, see Fig. Sm2. This region borders the study region of Lutz *et al.* (2014)[11] and that of Su *et al.* (2016)[13], where they studied the impacts of climate change on the hydrological regimes of the main rivers across Hindu-Kush-Himalayas ranges. By combining the studies together our aim is to draw up an overall picture of the impacts of climate change on hydrological regimes across the complete Tien Shan – Pamir – Karakoram and Himalayas ranges.

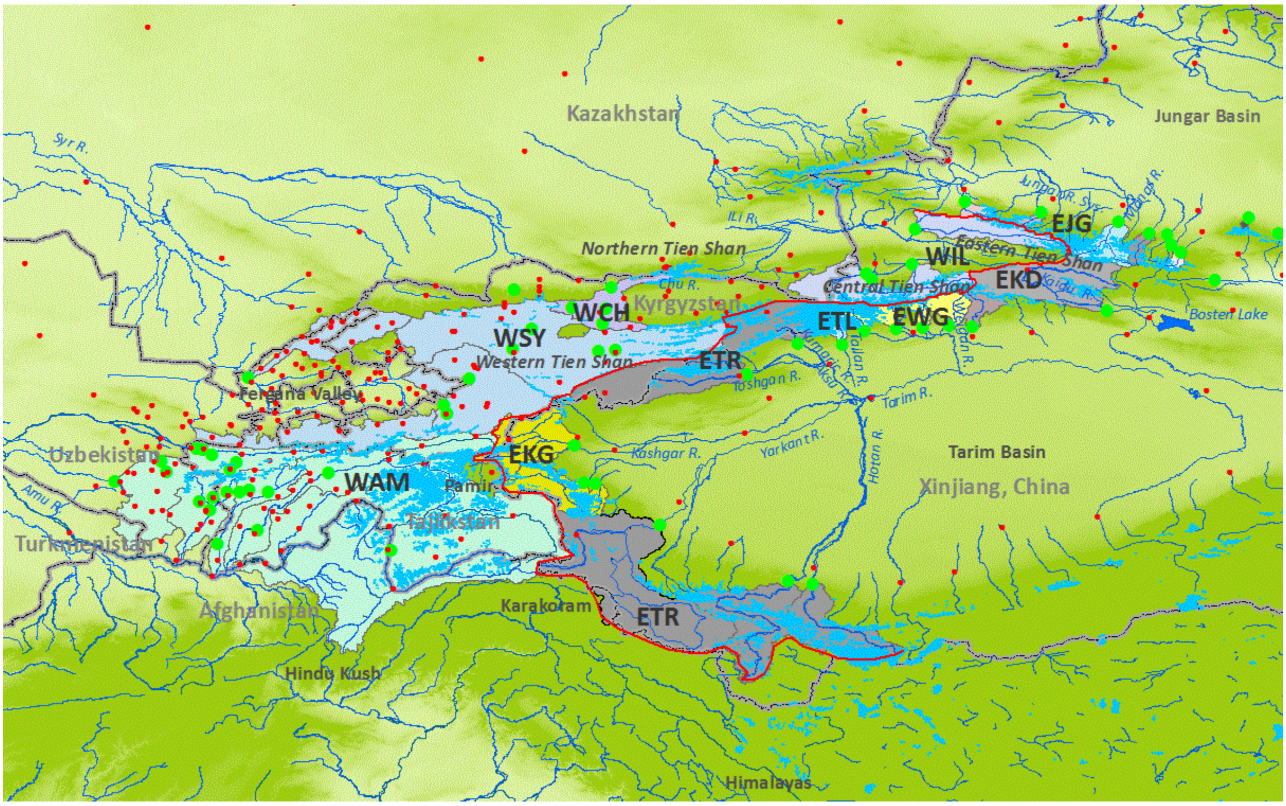

Fig. Sm2: a map depicting the locations of discharge gauges (green dots), meteorological stations (red dots) and glaciers (light blue patches) in the study area. On the eastern slope, the basins are: EJG, Jungar rivers; EKD, Kaidu River; EWG, Weigan River; ETL, Tailan River; ETR, Tarim River; EKG, Kashgar River; on the western slope, basins are: WIL, Ili River; WCH, Chu River; WSY, Syr River; and WAM, Amu River. The map was generated from the DEM data[22] (https://earthexplorer.usgs.gov/) on ArcGIS 9 ArcMap 9.3 (http://www.esri.com).

The physical features of the source regions of the ten main rivers in this study are listed in Table S1. The Syr Darya and Amu Darya on the west slope of the Tien Shan and Pamir are the main source rivers for the Aral Sea in Central Asia. The Chu River is an important water source of Kyrgyzstan. The Ili River originates in the Chinese territory of Xinjiang but then flows to Kazakhstan. Historically, the rivers around the Tarim Basin contribute to the main stream of the Tarim River. However, presently, only three main tributaries contribute to the main stream, namely, the Aksu River, the Yarkant River, and the Hotan River, which originate from the middle Tien Shan, the north slope of Karakoram, and the west Kunlun mountains, respectively; and the Aksu River contributes approximately three quarters of the streamflow[23,24].

3 Description of data

We used a digital elevation model (DEM-STRM3 with 90 m spatial resolution) to

delineate the source regions of the main rivers. Using ARCSWAT (http://swat.tamu.edu/software/arcswat/), we derived the outlines of the source regions, delineated the catchments into sub-basins and generated river systems based on the DEM.

Land-cover data were derived from the Globcover land-cover maps of the European Space Agency, which have 300 m × 300 m resolution.

For the Eastern and Central Tien Shan Mountains and Eastern Pamir and Karakoram Mountains, glacier data were compiled from the First Glacier Inventory (GIA – I) and the Second Glacier Inventory (GIA – II, Version 1.0) of China[20] and the Tien Shan Glaciers Dataset[21]. The GIA – I is for the period between the 1960s and 1970s, and GIA – II for the period 2007-2009. The Tien Shan Glacier Dataset includes two inventories: one for the 1970s and the other for the 2000s. The GIA – I and the Tien Shan Glaciers Datasets for the 1970s were compiled to initialize the simulation. In the western Tien Shan and Pamir Mountains, the glacier map has been merged from the Pamir Glacier Dataset V.1 (PGD)(http://www.asiacryoweb.org) as reported by Aizen *et al.*[25] and the World Glacier Inventory (WGI) released by the National Snow and Ice Data Center (NSID). The PGD databases contain the glacier area and the terminus elevation from the 1970s and the 2000s, respectively. Neither PGD nor WGI is complete in terms of the number of glaciers in the study area. These two databases were therefore compiled to generate a complete glacier database for the western Tien Shan and Pamir regions. We used glacier data sets for the 1960s and 1970s to initialize glacier coverage in the simulation (Fig. Sm2). We also used the compiled data sets to estimate glacier area changes between 2000s and 1960s. Literature data[26,27] was used as an additional source of information to estimate glacier area changes in the Amu Darya River basin.

Soil data were taken from the Harmonized World Soil Database (HWSD) of the Food and Agriculture Organization of the United Nations (http://www.fao.org/soils-portal/soil-survey/soil-maps-and-databases/harmonized-world-soil-database-v12/en/). Soil properties such as sand, silt and clay fractions, organic carbon and soil hydraulic

properties are included in the database. Other soil parameters such as available water capacity (AWC), hydraulic conductivity and moist bulk density required by the SWAT model are derived from the recorded soil attributes using the soil hydraulic property calculator SPAW[28] (https://hrsl.ba.ars.usda.gov/SPAW/SPAWDownload.html).

Ground-based meteorological observations are sparse in mountainous areas, especially in the study area, and are mainly available for valley areas. We collected daily data (Fig. Sm2) from the meteorological stations in the China section for the 1960s to 2000s and monthly data from the Central Asian section for the 1950s to the 1990s. In the Central Asia section, a number of the meteorological stations have been out of operation since the collapse of the Soviet Union in the late 1990s[29]. In addition to the ground observations, gridded databases exist that have continuous spatial coverage for a long time period, e.g., the APHRODITE[30] (the Asian Precipitation-Highly Resolved Observational Data Integration Towards Evaluation of Water Resources, http://www.chikyu.ac.jp/precip/english/products.html) and the PGMFD[31](Princeton Global Meteorological Forcing Dataset, http://hydrology.princeton.edu/data.pgf.php). The two data sets have been applied to glacio-hydrological simulation in the Tien Shan, Pamir, Karakoram, and Himalayas range in previous studies[11,32-35]. Daily precipitation data for the period 1951–2007 (with spatial resolution of 0.25° × 0.25°) were derived from APHRODITE. The daily maximum and minimum temperatures were collected from the PGMFD for 1948–2008 (with 0.5° × 0.5° spatial resolution). The temperature data were downscaled using a bi-linear interpolation method at a spatial resolution of 0.25° × 0.25°.

We evaluated the gridded precipitation and temperature data sets using the ground observations and derived the vertical lapse rates for estimating precipitation and temperature for elevations where data were not available.

At lower elevations where the observed precipitation data were available, we used them in

combination with APHRODITE precipitation data to estimate the precipitation lapse rates. At higher elevations with no glacier coverage, we estimated the precipitation lapse rate through the APHRODITE data. Using the precipitation lapse rates to drive the simulation, it was found that the precipitation was underestimated when we comprehensively checked the catchment water balance and the glacier area changes in the historical period. When we maintained the glacier area to change within a rational limit of observation, we found that the simulation underestimated the streamflow. When the simulation produced a reasonable estimation of the streamflow, the glacier area retreat was generally much more than the observations. Clarke *et al.*[5] bias-corrected the precipitation over glacier coverage. We followed another clue proposed by Kotlyakov and Krenke[36], who proposed a "global formula" to calculate the annual ablation at the Equilibrium Line Altitude (ELA):

$$A = 1.33 \times (9.66 + T_s)^{2.85} \tag{S10}$$

where A is the accumulation (or ablation) at the ELA per year (mm water equivalent) and T_s is the mean temperature in the summer (June–August) at the ELA (℃). It is assumed that accumulation or ablation at the ELA equals the precipitation with a multiplier 1.33 to account for the wind-blown snow and avalanches.

We simulated the glacier changes in late 1960s and early 1970s when the glacier area changes were minor. In the simulation, we adjusted the precipitation lapse rates so that the glaciers could maintain a neutral balance. We then calculated the precipitation at ELA of the glaciers following equation (S10). In a catchment, the ELA and the precipitation thereby of the glaciers varies with the altitude, and the precipitation lapse rate was derived as a result. Wang *et al.*[37] discussed this approach in detail, taking the Yarkent River catchment as an example. The same approach was applied to other catchments in this study as well.

We used the latest climate model ensemble generated for the fifth assessment report of the Intergovernmental Panel on Climate Change by the fifth phase of the Climate Model

Intercomparison Project (CMIP5) as the future climate forcing. In the simulation, we included three representative concentration pathways (RCPs) — RCP2.6, RCP4.5 and RCP8.5. The description of the GCM data management process is detailed in the next sections.

4 Description of climate and streamflow characteristics of the river basins

It is warm in summer in the source regions of the ten main rivers, with mean temperatures above 0 ℃ for May through September, and July is the warmest month. While the seasonal temperature distributions in the source regions are very similar, the seasonal precipitation distributions for the western and eastern slopes are different (Fig. Sm3, Table Sm1). On the western slopes, i.e., the Syr, Amu and Chu River source regions, it is wet from February to May (50% of the annual precipitation) and dry in July to September (only 10% of the annual precipitation). The only exception is the Ili River, which although it is on the western slope has a precipitation pattern similar to those on the east. In the source regions on the eastern slope, 66–78% of the annual precipitation falls in May to September. The Ili River basin is therefore discussed together with the eastern rivers hereafter. These rivers have very similar hydrographs, with distinctive wet and dry seasons. The rivers on the western slope have a high-flow season spanning from May to June, yet a high portion of the annual flow occurs during low flow conditions. On the eastern slope, the wet season is from June to August and low flows make little contribution. The higher low-flow proportion on the western slope is useful for power generation to meet wintertime demand in countries in the upstream region, e.g., Tajikistan and Kyrgyzstan. This, however, does not favour water demand in downstream regions which is mainly for irrigation water in the summer[38].

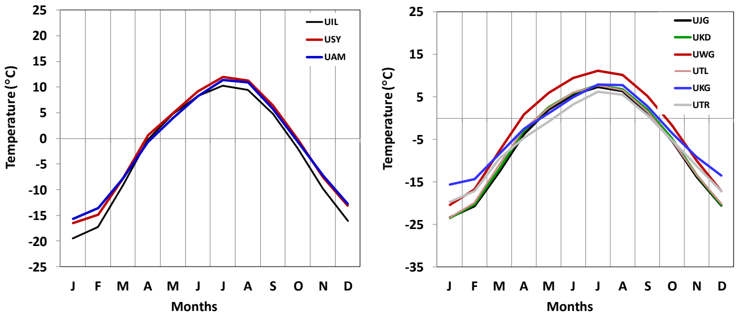

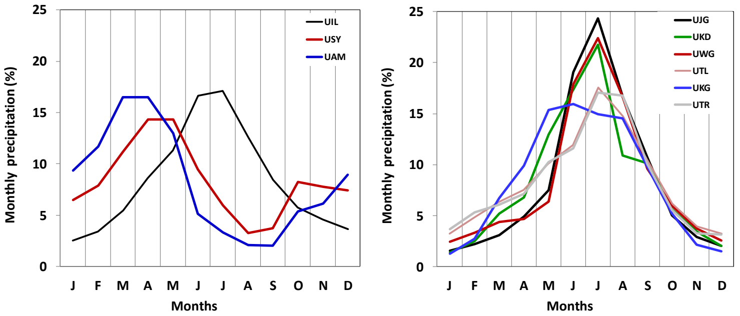

Fig. Sm3: monthly mean temperature and precipitation over 1966–1995 in source regions of the main rivers in western and eastern slopes of the Tien Shan – Pamir – Karakoram, respectively. See Fig. Sm2 for labelling of river catchments

Table Sm1: seasonal distribution of precipitation and streamflow of the major rivers in the Tien Shan-Pamir-Karakoram, averaged over 1966–1995. See Fig. Sm2 for labelling of river catchments

Month	Precipitation (mm)								
	WIL	WSY	WAM	EJG	EKD	EWG	ETL	EKG	ETR
1	17	37	53	7	7	11	21	4	10
2	22	45	66	10	12	16	31	9	15
3	36	63	94	13	25	20	41	21	17
4	57	82	93	22	32	22	49	31	20
5	75	82	73	33	61	30	66	48	28
6	110	54	29	84	82	84	77	50	32
7	113	34	19	107	103	105	114	47	47
8	84	19	12	73	52	78	96	46	46
9	56	21	12	47	48	45	67	31	28
10	38	47	31	22	27	28	40	16	15
11	30	44	35	13	16	17	26	7	9
12	24	42	51	9	10	12	21	5	9
sum	663	570	566	439	473	467	650	316	276
	Streamflow (mm)								
1	6	9	11	1	5	1	1	3	2
2	5	8	11	1	4	1	1	3	2
3	4	10	20	1	3	1	0	3	1
4	11	25	42	1	12	2	7	4	1
5	34	38	54	9	20	13	46	12	4
6	75	50	76	45	28	46	106	25	17
7	90	49	76	80	33	74	174	35	49
8	66	36	45	61	24	56	137	31	40
9	37	26	27	29	20	26	47	17	17
10	15	19	18	6	12	6	4	7	5
11	11	15	15	3	9	2	2	5	4
12	8	11	12	2	6	1	1	4	2
sum	362	296	405	239	177	228	527	150	145

5 *Glacio-hydrological model calibration and validation*

Basically, the streamflow record at each gauging station was set as the optimization target for the calibration and validation analyses. Additionally, glacier area change based on the two phases of the China Glacier Inventory and Tien Shan Glacier Dataset was used to constrain the calibration analysis. This was done to restrict over-parameterization due to the glacier melt compensation effect.

Discharge data are from China's Hydrological Yearbook, spanning the period 1961–1989. Data records after 1989 are not publically available. Discharge data at the gauges in Chu, Syr, and Amu River basins are from the Russian State Hydrological Institute (http://webworld.unesco.org/water) and the literature[39], spanning the 1960s–1990s and with gaps of missing data. Most of the discharge measurements are discontinuous since the 1990s. We split the data series into two segments for independent calibration and validation analysis. The catchments above the streamflow gauges are usually remote and scarcely influenced by human activities. The records are therefore considered as the natural state of the streamflow, except for that at Nurek, which is located downstream of a dam.

We use the Nash-Sutcliffe efficiency (*NSE*) and percent bias (*PBias*)[40] to evaluate the model performance during the calibration and validation periods. *NSE* and *PBias* are given as:

$$NSE = 1 - \frac{\sum_{i=1}^{n}(Q_i^{obs} - Q_i^{sim})^2}{\sum_{i=1}^{n}(Q_i^{obs} - Q^{mean})^2} \tag{S10}$$

$$PBias = \frac{\sum_{i=1}^{n}(Q_i^{obs} - Q_i^{sim}) \cdot 100}{\sum_{i=1}^{n} Q_i^{obs}} \tag{S11}$$

where Q_i^{obs} is the ith streamflow observation; Q_i^{sim} is the ith simulated streamflow; Q^{mean} is the mean observed streamflow; and n is the total number of observations.

NSE ranges between $-\infty$ and 1.0 (1.0 inclusive). NSE equals to unit being the optimal

value. When value of *NSE* is less than 0.0, it indicates that the mean observed value is better than the simulated value and the model performance is unacceptable. Values between 0.0 and 1.0 are generally viewed as acceptable levels of performance; the closer to the 1.0, the better the model performance is. Moriasi et al. rated the model performance on basis of the *NSE* values[40].

Moriasi *et al.*[40] rate model performance as 'very good', 'good', 'satisfactory' or 'unsatisfactory' if $0.75 < NSE \le 1.0$, $0.65 < NSE \le 0.75$, $0.50 < NSE \le 0.65$ or $NSE \le 0.50$, respectively; or if $PBias < \pm10\%$, $\pm10\% \le PBias < \pm15\%$, $\pm15\% \le PBias < \pm25\%$ or $PBias \ge \pm25\%$, respectively. We use rating system to evaluate the model performance.

For the parameterization of glaciated basins, the calibration could be wrong if the model is tuned with runoff measurements as the only target function. Inclusion of the glacier mass balance and snow measurements as additional criteria significantly reduces parameter uncertainty[29]. Given that the availability of glacier mass balance measurements are present for only limited benchmark glaciers, using the glacier area change based on different phases of glacier data as an additional criterion may help constrain parameter uncertainty during the calibration. We split the streamflow data series for each catchment into two independent segments, one for the model calibration and another for validation. For the glacier area change, however, the independent calibration and validation are hard to achieve because there are only two phases of glacier inventory data sets available.

The procedure for the parameter calibration is as follows: (1) adjust glacier parameters and check *NSE* and *PBias* at calibration stage; (2) run model through the period 1961–2007 and check simulated glacier area change against observed value. If the simulated value matches the observed one well, then (3) adjust other parameters to improve *PBias*. If not, then go back to (1) and re-adjust glacier parameters. This loop is repeated until *NSE*, *PBias* and glacier area change are acceptable. We manually calibrated the model by trial-and-error and monitored both the evaluation indices and internal processes during the calibration and validation periods. The processes such as

glacier and snow melt, glacier area change, streamflow processes, water balance, etc. are monitored during each simulation to ensure that the simulation processes, water balance and glacier area change are rational. The 'transparent' parameterization procedure ensures an efficient and reliable calibration. Model parameterization is crucial for model calibration and performance evaluation. Table Sm2 lists the most sensitive parameters for the glacier-enhanced SWAT model in previous studies in the region[4,34,41,42] and similar studies in other regions[43] along with the final parameters used in this study.

Table Sm2: main sensitive parameters calibrated in the glacier-enhanced SWAT model for the 24 catchments in the Tien Shan mountains (Reference range values are adopted from literatures[4,34,42,44,45])

Variable	Description	Unit	Reference range	Calibration results
$\delta_{gw, sh}$	Delay time of shallow aquifer	days	15 – 45	15 – 35
$\alpha_{gw,sh}$	Recession constant of shallow aquifer	—	0.15 – 0.4	0.02 – 0.8
$\delta_{gw,dep}$	Delay time of deep aquifer	days	15 – 127	100 – 150
$\alpha_{gw,dp}$	Recession constant of deep aquifer	—	0.02 – 0.05	0.01 – 0.08
SMTMP	Snowmelt base temperature	℃	0.5 – 1.5	0.5
SMFMX	Snowmelt factor for 21 June	mm ℃$^{-1}$ day^{-1}	1 – 6	2.5 – 4.5
SMFMN	Snowmelt factor for 21 December	mm ℃$^{-1}$ day^{-1}	1.0 – 3.5	1.0 – 3.5
GMTMP	Ice melt base temperature	℃	0.5 – 1.5	0.5
GMFMX	Ice melt factor for 21 June	mm ℃$^{-1}$ day^{-1}	2.0 – 12.8	2.1 – 27.3
GMFMN	Ice melt factor for 21 December	mm ℃$^{-1}$ day^{-1}	1 – 8	0.9 – 10.8

Fig. Sm4 depicts the *NSE* and *PBias* indices, Fig. Sm5 the regression analysis of the mean monthly simulated and observed discharge, Fig. Sm6 the comparison of simulated streamflow to the observations, and Fig. Sm7 the glacier area change. Most of the values suggest a 'very good' or 'good' parameterization. The *NSE* for the Nurek is 0.33, which is due to the influences of the Nurek dam. The Nurek dam was built in 1980, after which the discharge data at the Nurek station was not converted into its natural state. Thus, the simulated natural discharge process does not match the observed controlled process. Nevertheless, the *PBias* of – 5% suggested that the simulated discharge did not deviate seriously from the real situation in terms of inter-annual water balance[35]. The parameter values are used to project future glacio-hydrological changes in the study area.

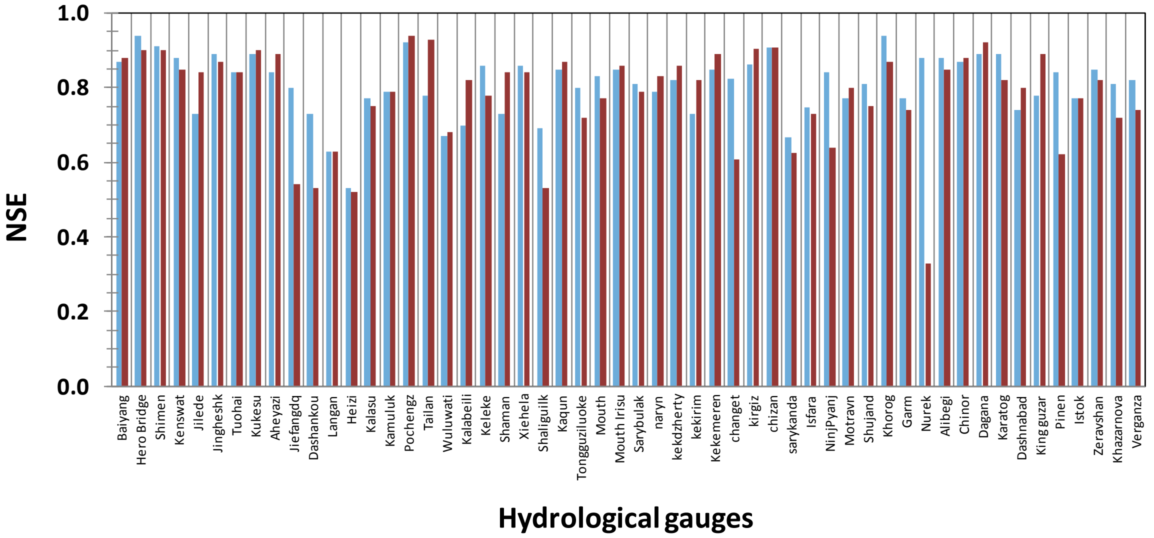

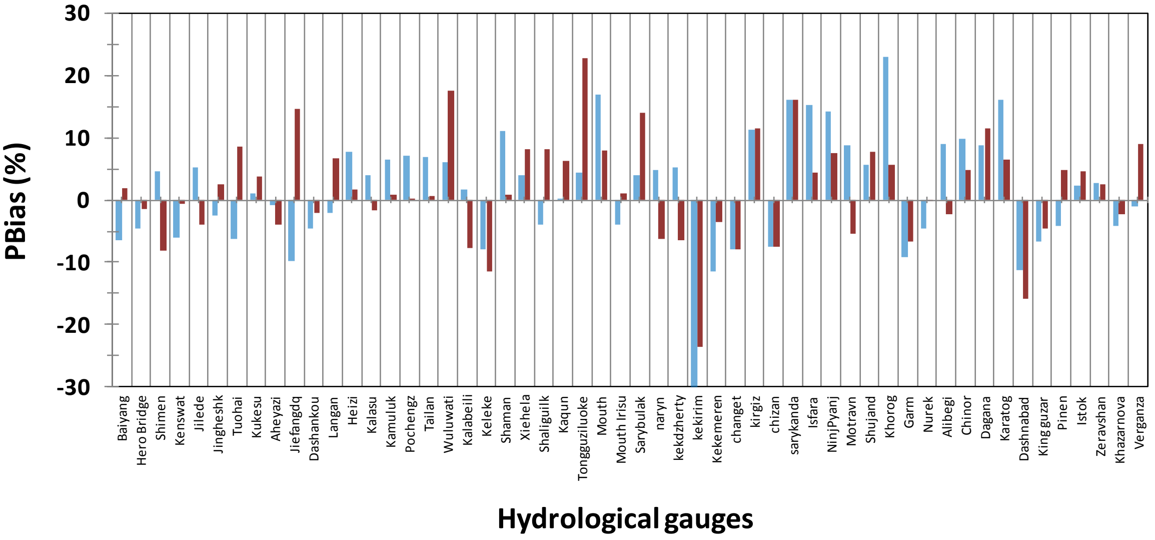

Fig. Sm4: the *NSE* (top) and *PBias* (bottom) evaluation indices for comparison of the simulated and observed monthly discharge at gauging stations in the source regions of the main rivers in the Tien Shan-Pamir-Karakoram(blue column for calibration and brown column for validation)

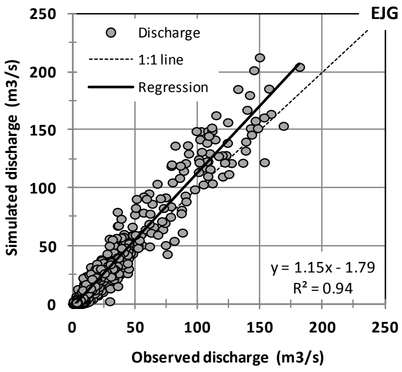

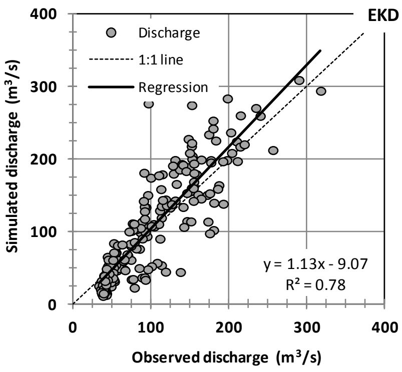

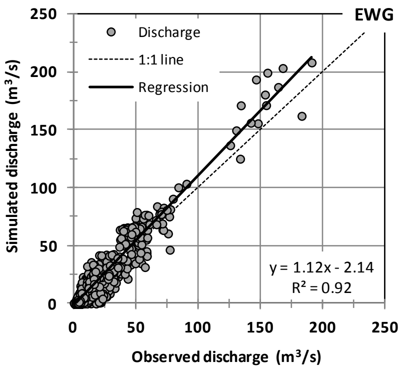

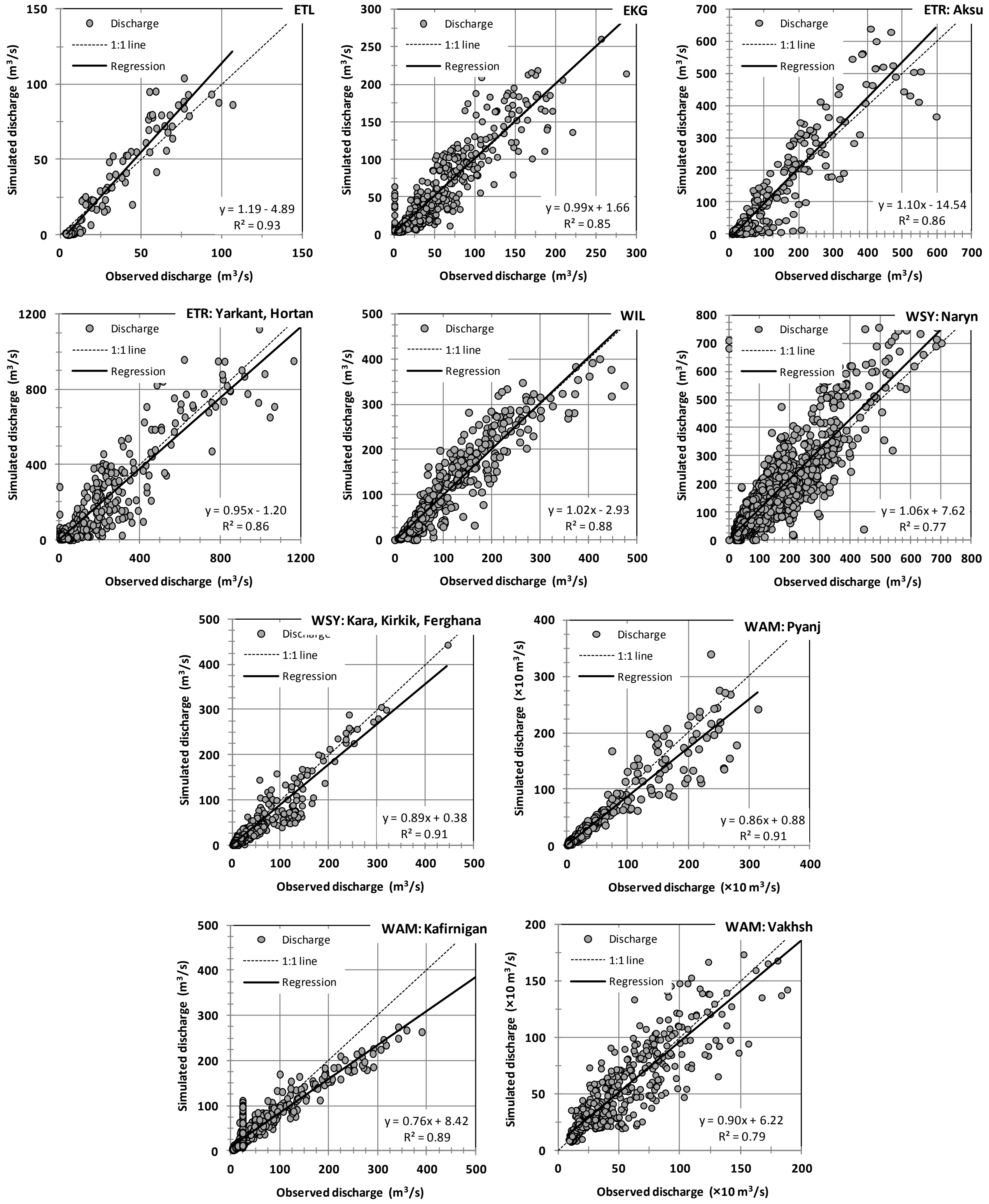

Fig. Sm5: regression analysis for the simulated and observed discharge at gauging stations in the main river basins in the Tien Shan-Pamir-Karakoram. A total of 54 gauges were used in this study. For clarity, the gauges are grouped on the basis of the main rivers or main tributaries in the study area. See Fig. Sm2 for labelling of river catchments

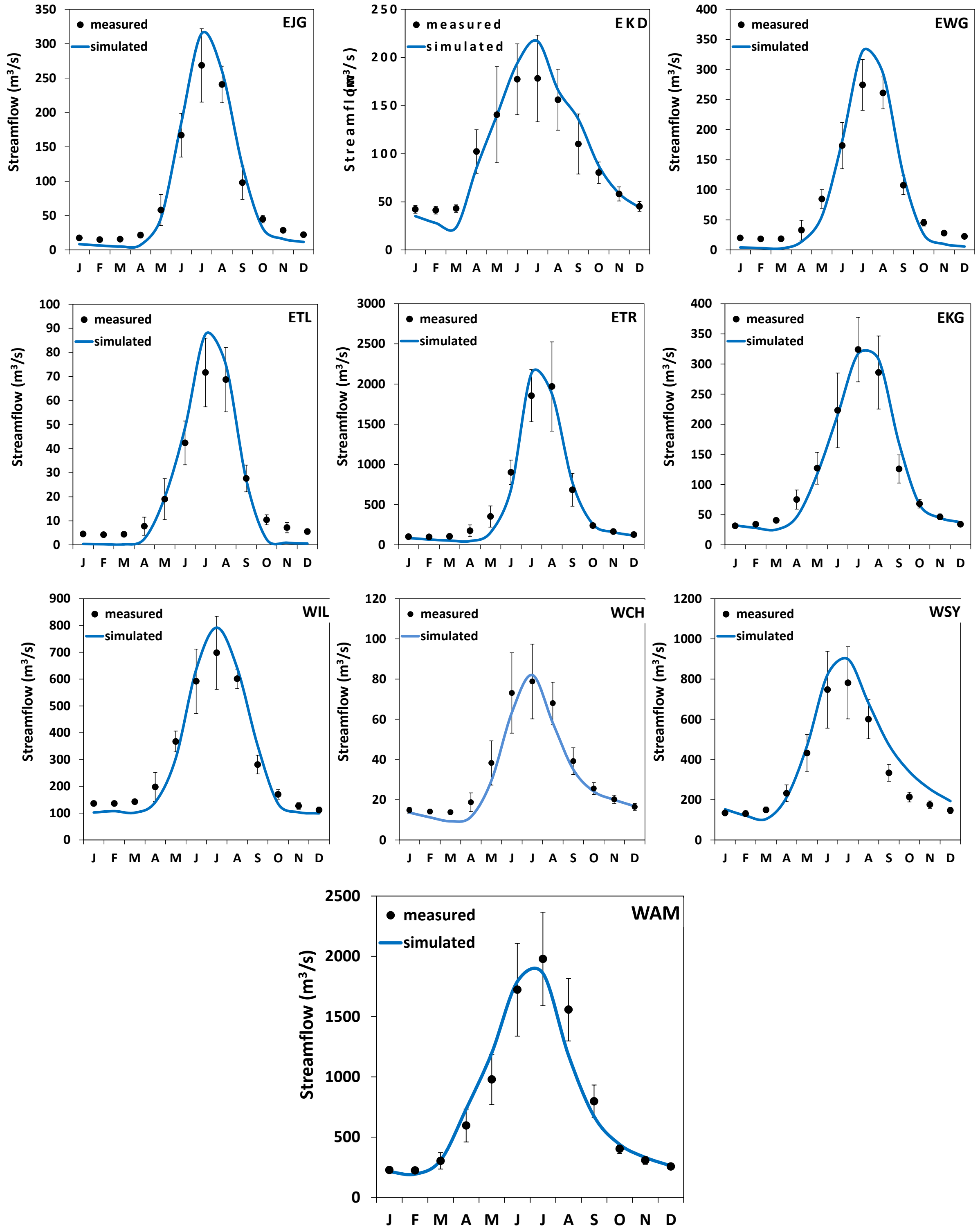

Fig. Sm6: comparisons of simulated and observed streamflow (with error bars showing the standard deviation) for the ten main rivers in the Tien Shan – Pamir – Karakoram. The monthly streamflow values are averaged over the period 1961-1989 for the eastern slope rivers, EJG (Jungar river system), EKD (Kaidu River), EWG (Weigan River), ETL (Tailan Rvier), ETR (Tarim Rvier), EKG (Kashgar River), and the western slope river, WIL (Ili River); the other western slope rivers, WCH (Chu River), WSY (Syr River), and WAM (Amu River) are averaged over the period 1961-1985

We constrain the parameterization of the glaciers by comparing the simulated and observed glacier area changes between two phases for the catchments (see SI Table S1) using the

data sets and literature data as mentioned in the section on description of data. Finally, the calibration achieved a very good match of the simulated glacier area changes to the observed ones in the benchmark catchments, Fig. Sm7 (left).

We compiled the glacier area in the benchmark catchments on basis of the Randolph Glacier Inventory[46] (RGI 5.0 available at http://www.glims.org/RGI/). We found that the RGI 5.0 records the glaciers in the benchmark catchments in 1998–2002 or 2007–2011. We compared simulated glacier area in 2000 or 2007 (end year of the simulation), to the group 1998–2002 or 2007–2011 of RGI 5.0, respectively. We found that they matched astonishingly well, Fig. Sm7 (right). Note that the RGI[46] and the glacier data we used in the simulation were not from the same data source.

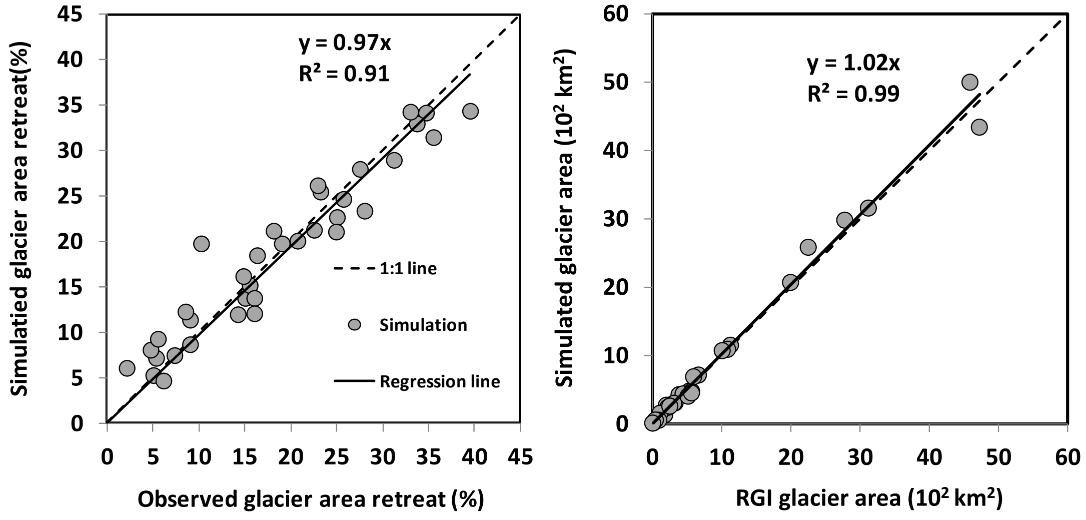

Fig. Sm7: comparison of simulated glacier area change to observed change in benchmark catchments in the Tien Shan – Pamir – Karakoram

6 Climate change and hydrological response simulation

6.1 Selection of GCM runs

Our model simulation was forced with the CMIP5 multi-model ensemble[47], the set of global climate change simulations used as the basis for the fifth assessment report of the Intergovernmental Panel on Climate Change (IPCC). We analysed the projected changes in average temperature (℃) and precipitation (%) between 1961–1990 and 2021–2050 for all available CMIP5 simulations for three representative concentration pathways (RCPs) — RCP2.6 (model runs),

RCP4.5 (model runs) and RCP8.5 (model runs). Based on the projected differences, four combinations (dry-and-cold, dry-and-warm, wet-and-cold, and wet-and-warm) were derived for each RCP at the 10th and 90th percentiles of the projected changes[11]. The model runs closest to the percentiles were eventually selected (Fig. Sm8, Table Sm3).

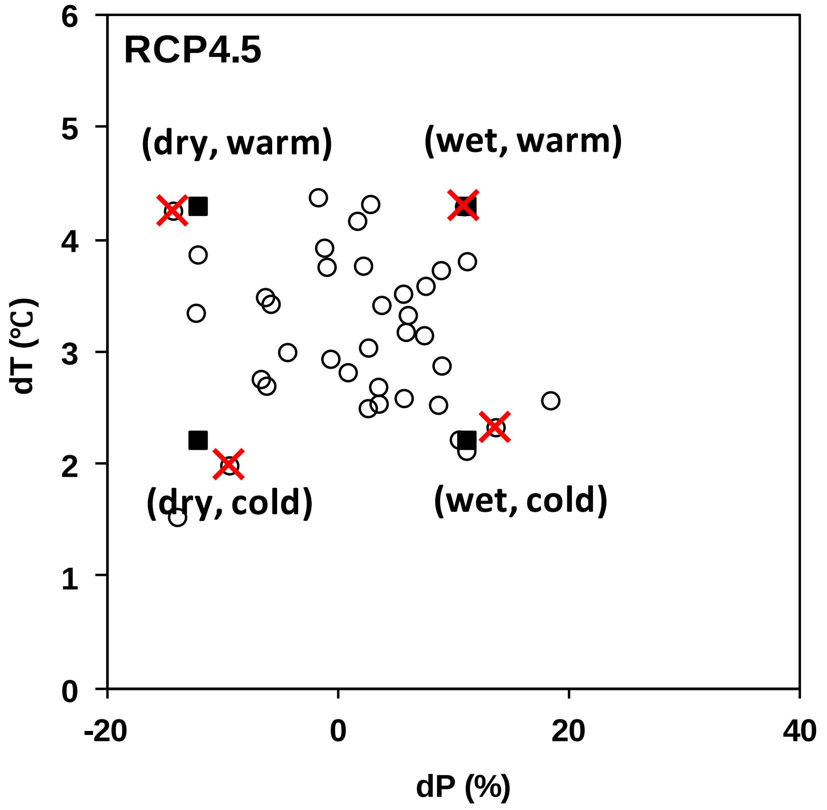

Fig. Sm8: an example of GCM run selection: climate change projection for the source region of Amu River under RCP4.5. Abbreviations: dP, relative difference between mean precipitation in 2066–2095 and 1966–1995; dT, absolute difference between mean temperatures in 2066–2095 and 1966–1995. Circles indicate GCM runs; solid boxes the 10% and 90% percentiles; and the crosses indicate the selected GCMs runs

Table Sm3: lists selected GCM runs for climate projection in source regions of the main rivers in the Tien Shan-Pamir-Karakoram

Sub-region (River)	RCP	P and T combination	Ensemble GCM member	dP (%)	dT (℃)
Eastern and Central Tien Shan EJG, EKD, EWG, ETL, WIL, and ETR: Akesu River	RCP2.6	Dry, Cold	MPI-ESM-MR	0.76	1.29
		Dry, Warm	NorESM1-M	-0.94	2.75
		Wet, Cold	EC-EARTH	11.97	1.33
		Wet, Warm	MIROC-ESM	16.21	3.18
	RCP4.5	Dry, Cold	inmcm4	0.08	1.52
		Dry, Warm	HadGEM2-ES	-4.03	3.87
		Wet, Cold	CNRM-CM5	21.94	2.19
		Wet, Warm	MIROC-ESM	17.37	4.39
	RCP8.5	Dry, Cold	inmcm4	-0.98	3.71

		Dry, Warm	HadGEM2-ES	-6.35	6.24
		Wet, Cold	CNRM-CM5	21.79	4.14
		Wet, Warm	MIROC-ESM	16.80	7.53
Western Tien Shan WCH, WSY	RCP2.6	Dry, Cold	bcc-csm1-1	-0.54	1.56
		Dry, Warm	IPSL-CM5A-LR	-6.20	2.66
		Wet, Cold	MPI-ESM-LR	12.45	1.19
		Wet, Warm	CanESM2	13.82	2.95
	RCP4.5	Dry, Cold	FIO-ESM	-7.13	1.41
		Dry, Warm	IPSL-CM5A-LR	-9.60	3.80
		Wet, Cold	MRI-CGCM3	18.57	2.18
		Wet, Warm	CanESM2	12.46	4.09
	RCP8.5	Dry, Cold	inmcm4	-6.37	3.79
		Dry, Warm	IPSL-CM5A-LR	-12.00	6.48
		Wet, Cold	CNRM-CM5	17.50	4.58
		Wet, Warm	CanESM2	19.12	6.96
Pamir WAM	RCP2.6	Dry, Cold	bcc-csm1-1	-3.21	1.70
		Dry, Warm	IPSL-CM5A-LR	-5.19	2.88
		Wet, Cold	EC-EARTH	10.05	1.49
		Wet, Warm	CanESM2	14.17	3.08
	RCP4.5	Dry, Cold	inmcm4	-9.31	1.99
		Dry, Warm	IPSL-CM5A-LR	-14.18	4.26
		Wet, Cold	MRI-CGCM3	13.76	2.33
		Wet, Warm	CanESM2	11.03	4.30
	RCP8.5	Dry, Cold	FIO-ESM	-23.72	4.05
		Dry, Warm	IPSL-CM5A-LR	-24.88	7.04
		Wet, Cold	MRI-CGCM3	17.57	4.64
		Wet, Warm	CanESM2	17.15	7.11
Eastern Pamir EKG	RCP2.6	Dry, Cold	MPI-ESM-MR	-10.87	1.63
		Dry, Warm	IPSL-CM5A-LR	-4.24	2.63
		Wet, Cold	CESM1-WACCM	10.65	1.35
		Wet, Warm	MROC5	22.07	2.25
	RCP4.5	Dry, Cold	inmcm4	-4.75	1.70
		Dry, Warm	IPSL-CM5A-LR	-13.65	3.80
		Wet, Cold	CNRM-CM5	14.16	2.37
		Wet, Warm	MROC5	23.64	3.30
	RCP8.5	Dry, Cold	MPI-ESM-MR	-19.74	5.08
		Dry, Warm	IPSL-CM5A-LR	-26.51	6.58
		Wet, Cold	CNRM-CM5	20.67	3.95
		Wet, Warm	MROC5	34.73	5.23
Karakoram ETR: Yarkant River and Hortan River	RCP2.6	Dry, Cold	MPI-ESM-MR	0.09	1.49
		Dry, Warm	IPSL-CM5A-LR	0.99	2.87
		Wet, Cold	CESM1-WACCM	18.05	1.04
		Wet, Warm	CanESM2	18.11	2.91
	RCP4.5	Dry, Cold	FIO-ESM	-1.13	1.72

	Dry, Warm	IPSL-CM5A-LR	-0.92	4.44
	Wet, Cold	MRI-CGCM3	21.28	2.32
	Wet, Warm	CanESM2	21.17	4.05
RCP8.5	Dry, Cold	FIO-ESM	-9.20	4.41
	Dry, Warm	IPSL-CM5A-LR	-1.10	7.46
	Wet, Cold	CNRM-CM5	33.68	3.95
	Wet, Warm	CanESM2	34.99	6.68

Abbreviations: P, precipitation; T, temperature; dP, relative difference between averaged precipitation for 2066–2095 and 1966–1995; dT, absolute difference between averaged temperature for 2066–2095 and 1966–1995. See Fig. Sm2 for labelling of river catchments

6.2 Downscaling and hydrological simulation

We used the 'delta change' approach[11,48-50] to downscale the selected GCM outputs, generating the data series to force simulation of future hydrologic processes. The downscaling includes three steps[11,50] (1) estimation of mean values of each GCM simulated baseline and future climate; (2) calculation of additive change factors for temperature and multiplicative change factors for precipitation; and (3) application of the change factors (temperature and precipitation) to local values to get future local-scale values.

Based on the gridded 0.25 °-resolution meteorological data set for 1950–2005, we found the nearest point from the GCM grid cells and computed a transient “delta change” value. We repeated this procedure for each selected GCM run and for each month, taking into account seasonal differences in the GCM output. We then selected a random year from the historical database and superimposed the monthly temperature and precipitation change grids to construct a transient time series from 2006–2100.

We used the generated time series of temperature and precipitation to force the glacier-enhanced SWAT model to simulate the hydrological processes for 1961–2100. To determine the impact of climate change on future hydrological regimes in the source regions of the main rivers, we compared the ensemble averages of the simulated hydrological outputs for 1966–1995, 2016–2045 and 2066–2095. We used this to determine the long-term patterns of change in the hydrological regimes and water availability in each river under each climate change scenario. The

ensemble members of the GCMs represent the scope of uncertainties of the climatic input. The hydrological outputs of each GCM model run give the scope of uncertainties in hydrological regimes and water availability of the main rivers.

6.3 Projected temperature and precipitation change in the future

We used the output of the latest GCM ensemble to project future changes in streamflow and streamflow components. For each Representative Concentration Pathway (RCP), we selected four GCM runs with projections of wet-and-warm, wet-and-cold, dry-and-warm or dry-and-cold, representing the range of possible future conditions. The ensemble means for the glacio-hydrological simulations, forced by respective GCM runs, give the general trends and uncertainties in future hydrological regimes and water availability. These GCM runs were downscaled to reconstruct transient daily time series of precipitation and temperature up to 2100. The ensemble members of the GCMs suggest a consistent increase in temperature under all RCPs. Relative to the reference period 1966–1995, the projected increase in temperature is 1.4–2.2 °C for the near future (2016–2045) and 2.2–6.0 °C for the distant future (2066–2095), with significant variations in RCPs and marginal differences in river basins (Fig. Sm9, Table Sm4).

The ensemble mean of the GCM runs projects a generally wetter trend for both the western and eastern slopes of the TPK. Under the three RCP conditions, the GCMs foresee precipitation change of -3.7 to +7.8% in 2016–2045 and -1.7 to +9.8% in 2066–2095, relative to 1966–1995. There is only a decrease in precipitation in 2016–2045 in source regions of Chu River under RCP2.6 and RCP4.5 and Syr River under RCP4.5, then in 2066–2095 in the source region of Amu River under RCP8.5. Precipitation change is marginal in Amu, Syr and Chu River basins, while it is remarkable in the other river basins. The difference in precipitation between the western and eastern slopes is due to the high mountain ranges whereas the spatial heterogeneity is due to the local topography. While the northern Tien Shan Mountains block atmospheric moisture from reaching Chu River basin, the western horn-shaped Ili River basin routinely receives moisture. The highly heterogeneous precipitation and glacier cover, the changes in precipitation and glacier cover,

and the combined effects of precipitation and glacier cover complicate any analysis of the hydrological regime in the Tien Shan-Pamir-Karakoram under climate change.

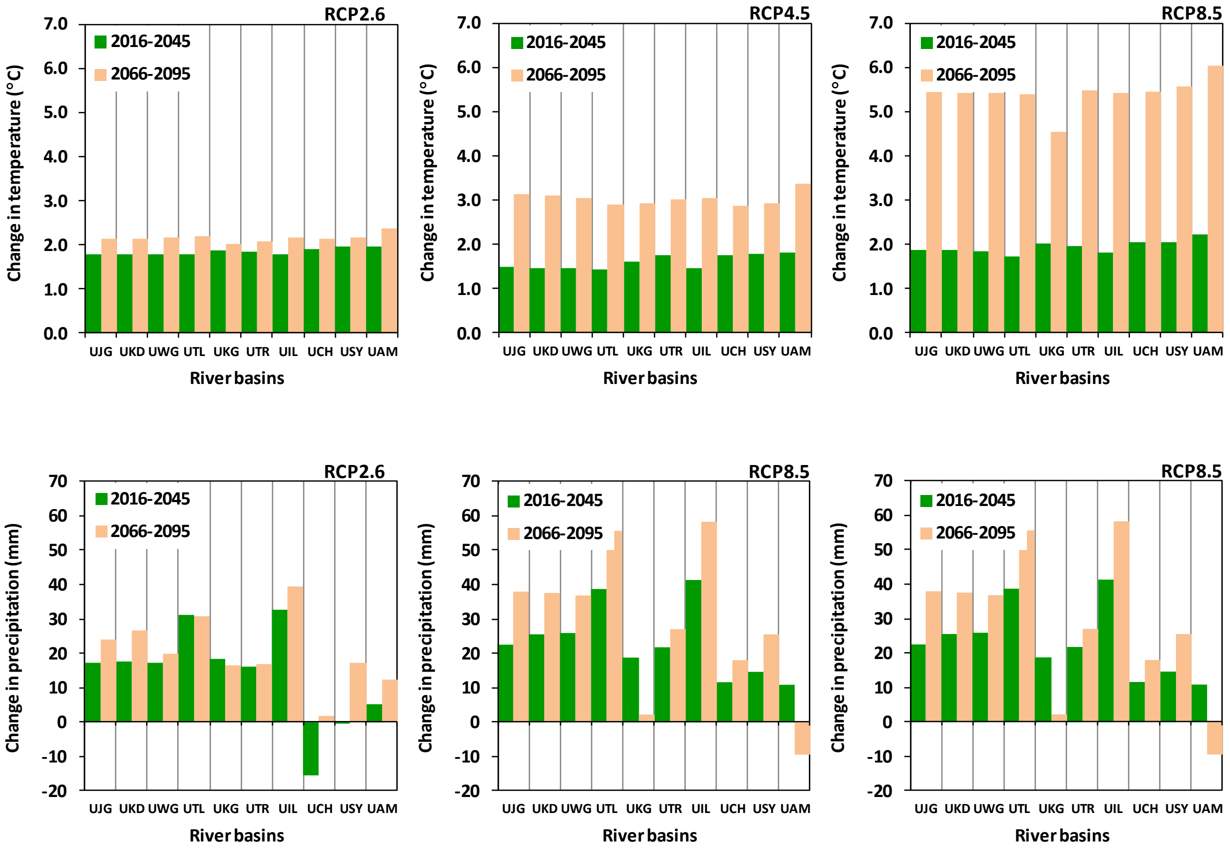

Fig. Sm9: projected changes in temperature (top) and precipitation (bottom) relative to 1966–1995 in the upstream river basins in the Tien Shan-Pamir-Karakoram. See Fig. Sm2 for labelling of river catchments

Table Sm4: projections of the temperature and precipitation changes in source regions of the main rivers in the Tien Shan-Pamir-Karakoram. See Fig. Sm2 for labelling of river catchments

Rivers	Reference	RCP2.6		RCP4.5		RCP8.5	
	1966-1995	2016-2045	2066-2095	2016-2045	2066-2095	2016-2045	2066-2095
	(℃)	Changes in temperature (℃)					
EJG	-6.39	1.8	2.1	1.5	3.1	1.9	5.5
EKD	-5.93	1.8	2.1	1.5	3.1	1.9	5.4
EWG	-2.49	1.8	2.2	1.5	3.1	1.8	5.4
ETL	-5.92	1.8	2.2	1.4	2.9	1.7	5.4
EKG	-3.49	1.9	2.0	1.6	2.9	2.0	4.5
ETR	-5.76	1.8	2.1	1.7	3.0	2.0	5.5
WIL	-2.96	1.8	2.2	1.5	3.0	1.8	5.4
WCH	-2.21	1.9	2.1	1.7	2.9	2.1	5.5
WSY	-1.24	2.0	2.2	1.8	2.9	2.0	5.6

WAM	-1.48	2.0	2.4	1.8	3.4	2.2	6.0
min	-6.4	1.8	2.0	1.4	2.9	1.7	4.5
max	-1.2	2.0	2.4	1.8	3.4	2.2	6.0
	(mm)	Relative changes in precipitation (%)					
EJG	489	3.5	4.9	3.9	7.5	4.6	7.7
EKD	473	3.7	5.6	5.1	7.9	5.4	7.9
EWG	467	3.7	4.3	6.1	8.6	5.6	7.9
ETL	650	4.8	4.7	6.6	8.5	6.0	8.6
EKG	316	5.8	5.2	5.7	3.6	6.0	0.7
ETR	277	5.9	6.1	7.7	9.7	7.8	9.8
WIL	663	4.9	5.9	6.1	9.2	6.3	8.8
WCH	426	-3.7	0.4	-0.7	3.4	2.7	4.2
WSY	569	-0.1	3.0	2.2	4.8	2.6	4.5
WAM	567	0.9	2.2	1.7	0.9	1.9	-1.7
min		-3.7	0.4	-0.7	0.9	1.9	-1.7
max		5.9	6.1	7.7	9.7	7.8	9.8

7 References

1 Jiang, X., Wang, N., He, J., Wu, X. & Song, G. A distributed surface energy and mass balance model and its application to a mountain glacier in China. *Chinese Science Bulletin* **55**, 2079-2087, doi:10.1007/s11434-010-3068-9 (2010).

2 Hock, R. Temperature index melt modelling in mountain areas. *Journal of Hydrology* **282**, 104-115, doi:10.1016/s0022-1694(03)00257-9 (2003).

3 Anderson, E. A. National weather service river forecast system/snow accumulation and ablation model. *NOAA Technical Memorandum NWS HYDRO-17*, 217 (1973).

4 Luo, Y., Arnold, J., Liu, S., Wang, X. & Chen, X. Inclusion of glacier processes for distributed hydrological modeling at basin scale with application to a watershed in Tianshan Mountains, northwest China. *Journal of Hydrology* **477**, 72-85, doi:10.1016/j.jhydrol.2012.11.005 (2013).

5 Clarke, G. K. C., Jarosch, A. H., Anslow, F. S., Radic, V. & Menounos, B. Projected deglaciation of western Canada in the twenty-first century. *Nature Geoscience* **8**, 372-377, doi:10.1038/ngeo2407 (2015).

6 Chen, J., Ohmura, A. Estimation of Alpine glacier water resources and their change since 1870s. 9 (1990).

7 Raper, S. C. B. & Braithwaite, R. J. Low sea level rise projections from mountain glaciers and icecaps under global warming. *Nature* **439**, 311-313, doi:10.1038/nature04448 (2006).

8 Radic, V. & Hock, R. Regionally differentiated contribution of mountain glaciers and ice caps to future sea-level rise. *Nature Geoscience* **4**, 91-94, doi:10.1038/ngeo1052 (2011).

9 Harrison, W. D. How do glaciers respond to climate? Perspectives from the simplest models. *Journal of Glaciology* **59**, 949-960, doi:10.3189/2013JoG13J048 (2013).

10 Immerzeel, W. W., Pellicciotti, F. & Bierkens, M. F. P. Rising river flows throughout the twenty-first century in two Himalayan glacierized watersheds. *Nature Geoscience* **6**, 742-745, doi:10.1038/ngeo1896 (2013).

11 Lutz, A. F., Immerzeel, W. W., Shrestha, A. B. & Bierkens, M. F. P. Consistent increase in High Asia's runoff due to increasing glacier melt and precipitation. *Nature Climate Change* **4**, 587-592, doi:10.1038/nclimate2237 (2014).

12 Ragettli, S., Immerzeel, W. W. & Pellicciotti, F. Contrasting climate change impact on river flows from high-altitude catchments in the Himalayan and Andes Mountains. *Proceedings of the National Academy of Sciences of the United States of America* **113**, 9222-9227, doi:10.1073/pnas.1606526113 (2016).

13 Su, F., Zhang, L., Ou, T., Chen, D., Yao, T., Tong, K. & Qi, Y. Hydrological response to future climate changes for the major upstream river basins in the Tibetan Plateau. *Global and Planetary Change* **136**, 82-95, doi:10.1016/j.gloplacha.2015.10.012 (2016).

14 Zhao, Q., Zhang, S., Ding, Y., Wang, J., Han, H., Xu, J., Zhao, C., Guo, W. & Shangguan, D. Modeling Hydrologic Response to Climate Change and Shrinking Glaciers in the Highly Glacierized Kunma Like River Catchment, Central Tian Shan. *Journal of Hydrometeorology* **16**, 2383-2402, doi:10.1175/jhm-d-14-0231.1 (2015).

15 Hirabayashi, Y., Doell, P. & Kanae, S. Global-scale modeling of glacier mass balances for water resources assessments: Glacier mass changes between 1948 and 2006. *Journal of Hydrology* **390**, 245-256, doi:10.1016/j.jhydrol.2010.07.001 (2010).

16 Bahr, D. B., Pfeffer, W. T. & Kaser, G. A review of volume-area scaling of glaciers. *Reviews of Geophysics* **53**, 95-140, doi:10.1002/2014rg000470 (2015).

17 Huss, M. & Farinotti, D. Distributed ice thickness and volume of all glaciers around the globe. *Journal of Geophysical Research-Earth Surface* **117**, doi:10.1029/2012jf002523 (2012).

18 Liu, S. Y., Sun, W. X., Shen, Y. P. & Li, G. Glacier changes since the Little Ice Age maximum in the western Qilian Shan, northwest China, and consequences of glacier runoff for water supply. *Journal of Glaciology* **49**, 117-124 (2003).

19 Radic, V. & Hock, R. Glaciers in the Earth's Hydrological Cycle: Assessments of Glacier Mass and Runoff Changes on Global and Regional Scales. *Surveys in Geophysics* **35**, 813-837, doi:10.1007/s10712-013-9262-y (2014).

20 Arnold, J. G., Srinivasan, R., Muttiah, R. S. & Williams, J. R. Large area hydrologic modeling and assessment - Part 1: Model development. *Journal of the American Water Resources Association* **34**, 73-89, doi:10.1111/j.1752-1688.1998.tb05961.x (1998).

21 Neitsch, S. L., Arnold, J. G., Kiniry, J. R., Williams, J. R., King, K. W. Soil and water assessment tool theoretical documentation: version 2005. (2005).

22 Jarvis, J., Reuter, H., Nelson, A., Guevara, E. SRTM 90m Digital Elevation Data version 4 *CGIAR-CSI* (2008).

23 Ling, H. B., Guo, B., Xu, H. L. & Fu, J. Y. Configuration of water resources for a typical river basin in an arid region of China based on the ecological water requirements (EWRs) of desert riparian vegetation. *Global and Planetary Change* **122**, 292-304, doi:10.1016/j.gloplacha.2014.09.008 (2014).

24 Li, Z. H., Hao, Z. C., Shi, X. G., Dery, S. J., Li, J. Y., Chen, S. C. & Li, Y. K. An agricultural drought index to incorporate the irrigation process and reservoir operations: A case study in the Tarim River Basin. *Global and Planetary Change* **143**, 10-20, doi:10.1016/j.gloplacha.2016.05.008 (2016).

25 Aizen, V. B., Kuzmichenok, V. A., Surazakov, A. B. & Aizen, E. M. Glacier changes in the Tien Shan as determined from topographic and remotely sensed data. *Global and Planetary Change* **56**, 328-340, doi:10.1016/j.gloplacha.2006.07.016 (2007).

26 Konovalov, V. G. & Shchetinnicov, A. S. Evolution of glaciation in the Pamiro-Alai mountains and its effect on river run-off. *Journal of Glaciology* **40**, 149-157 (1994).

27 Konovalov, V. Past and perspective changes in the state of Central Asian glaciers. *Ice and Snow* **3(115)**, 9 (2011).

28 Saxton, K. E. & Rawls, W. J. Soil water characteristic estimates by texture and organic matter for hydrologic solutions. *Soil Science Society of America Journal* **70**, 1569-1578, doi:10.2136/sssaj2005.0117 (2006).

29 Sorg, A., Bolch, T., Stoffel, M., Solomina, O. & Beniston, M. Climate change impacts on glaciers and runoff in Tien Shan (Central Asia). *Nature Climate Change* **2**, 725-731, doi:10.1038/nclimate1592 (2012).

30 Yatagai, A., Kamiguchi, K., Arakawa, O., Hamada, A., Yasutomi, N. & Kitoh, A. APHRODITE Constructing a Long-Term Daily Gridded Precipitation Dataset for Asia Based on a Dense Network of Rain Gauges. *Bulletin of the American Meteorological Society* **93**, 1401-1415, doi:10.1175/bams-d-11-00122.1 (2012).

31 Sheffield, J., Goteti, G. & Wood, E. F. Development of a 50-year high-resolution global dataset of meteorological forcings for land surface modeling. *Journal of Climate* **19**, 3088-3111, doi:10.1175/jcli3790.1 (2006).

32 Immerzeel, W. W., van Beek, L. P. H., Konz, M., Shrestha, A. B. & Bierkens, M. F. P. Hydrological response to climate change in a glacierized catchment in the Himalayas. *Climatic Change* **110**, 721-736, doi:10.1007/s10584-011-0143-4 (2012).

33 Hagg, W., Hoelzle, M., Wagner, S., Mayr, E. & Klose, Z. Glacier and runoff changes in the Rukhk catchment, upper Amu-Darya basin until 2050. *Global and Planetary Change* **110**, 62-73, doi:10.1016/j.gloplacha.2013.05.005 (2013).

34 Gan, R., Luo, Y., Zuo, Q. & Sun, L. Effects of projected climate change on the glacier and runoff generation in the Naryn River Basin, Central Asia. *Journal of Hydrology* **523**, 240-251, doi:10.1016/j.jhydrol.2015.01.057 (2015).

35 Wang, X., Luo, Y., Sun, L., He, C., Zhang, Y. & Liu, S. Attribution of Runoff Decline in the Amu Darya River in Central Asia during 1951-2007. *Journal of Hydrometeorology* **17**, 1543-1560, doi:10.1175/jhm-d-15-0114.1 (2016).

36 Kotlyakov, V. M. & Krenke, A. N. Investigations of the hydrological conditions of alpine regions by glaciological methods. *Hydrological Sciences Journal-Journal Des Sciences Hydrologiques* **27**, 251-252 (1982).

37 Wang, X. L., Sun, L., Zhang, Y. Q. & Luo, Y. Rationalization of Altitudinal Precipitation Profiles in a Data-Scarce Glacierized Watershed Simulation in the Karakoram. *Water* **8**, doi:10.3390/w8050186 (2016).

38 Siegfried, T. *et al.* Will climate change exacerbate water stress in Central Asia? *Climatic Change* **112**, 881-899, doi:10.1007/s10584-011-0253-z (2012).

39 Kure, S., Jang, S., Ohara, N., Kavvas, M. L. & Chen, Z. Q. Hydrologic impact of regional climate change for the snowfed and glacierfed river basins in the Republic of Tajikistan: hydrological response of flow to climate change. *Hydrological Processes* **27**, 4057-4070, doi:10.1002/hyp.9535 (2013).

40 Moriasi, D. N., Arnold, J. G., Van Liew, M. W., Bingner, R. L., Harmel, R. D. & Veith, T. L. Model evaluation guidelines for systematic quantification of accuracy in watershed simulations. *Transactions of the Asabe* **50**, 885-900 (2007).

41 Luo, Y., Arnold, J., Allen, P. & Chen, X. Baseflow simulation using SWAT model in an inland river basin in Tianshan Mountains, Northwest China. *Hydrology and Earth System Sciences* **16**, 1259-1267, doi:10.5194/hess-16-1259-2012 (2012).

42 Ma, C., Sun, L., Liu, S., Shao, M. a. & Luo, Y. Impact of climate change on the streamflow in the glacierized Chu River Basin, Central Asia. *Journal of Arid Land* **7**, 501-513, doi:10.1007/s40333-015-0041-0 (2015).

43 Hagg, W., Braun, L. N., Kuhn, M. & Nesgaard, T. I. Modelling of hydrological response to climate change in glacierized Central Asian catchments. *Journal of Hydrology* **332**, 40-53, doi:10.1016/j.jhydrol.2006.06.021 (2007).

44 Gan, R. & Luo, Y. Using the nonlinear aquifer storage-discharge relationship to simulate the base flow of glacier- and snowmelt-dominated basins in northwest China. *Hydrology and Earth System Sciences* **17**, 3577-3586, doi:10.5194/hess-17-3577-2013 (2013).

45 Wang, X., Luo, Y., Sun, L. & Zhang, Y. Assessing the effects of precipitation and temperature changes on hydrological processes in a glacier-dominated catchment. *Hydrological Processes* **29**, 4830-4845, doi:10.1002/hyp.10538 (2015).

46 Pfeffer, W. T., Arendt, A. A., Bliss, A., Bolch, T., Cogley, J., Gardner, S., Hagen, J., Hock, R., Kaser, G., Kienholz, C., Miles, S., Moholdt, G., Moelg, N., Paul, F., Radic, V., Rastner, P., Raup, H., Rich, J., Sharp, J., Andeassen, L. M., Bajracharya, S., Barrand, N. E., Beedle, M. J., Berthier, E., Bhambri, R., Brown, I., Burgess, D. O., Burgess, E. W., Cawkwell, F., Chinn, T., Copland, L., Cullen, N. J., Davies, B., De Angelis, H., Fountain, A. G., Frey, H., Giffen, B. A., Glasser, N. F., Gurney, S. D., Hagg, W., Hall, D. K., Haritashya, U. K., Hartmann, G., Herreid, S., Howat, I., Jiskoot, H., Khromova, T. E., Klein, A., Kohler, J., Konig, M., Kriegel, D., Kutuzov, S., Lavrentiev, I., Le Bris, R., Li, X., Manley, W. F., Mayer, C., Menounos, B., Mercer, A., Mool, P., Negrete, A., Nosenko, G., Nuth, C., Osmonov, A., Pettersson, R., Racoviteanu, A., Ranzi, R., Sarikaya, M. A., Schneider, C., Sigurdsson, O., Sirguey, P., Stokes, C. R., Wheate, R., Wolken, G. J., Wu, L. Z., Wyatt, F. R. & Randolph, Consortium. The Randolph Glacier Inventory: a globally complete inventory of glaciers. *Journal of Glaciology* **60**, 537-552, doi:10.3189/2014JoG13J176 (2014).

47 Taylor, K. E., Stouffer, R. J. & Meehl, G. A. AN OVERVIEW OF CMIP5 AND THE EXPERIMENT DESIGN. *Bulletin of the American Meteorological Society* **93**, 485-498, doi:10.1175/bams-d-11-00094.1 (2012).

48 Arnell, N. W. Climate change and global water resources. *Global Environmental Change-Human and Policy Dimensions* **9**, S31-S49, doi:10.1016/s0959-3780(99)00017-5 (1999).

49 Prudhomme, C., Reynard, N. & Crooks, S. Downscaling of global climate models for flood frequency analysis: where are we now? *Hydrological Processes* **16**, 1137-1150, doi:10.1002/hyp.1054 (2002).

50 Kay, A. L., Davies, H. N., Bell, V. A. & Jones, R. G. Comparison of uncertainty sources for climate change impacts: flood frequency in England. *Climatic Change* **92**, 41-63, doi:10.1007/s10584-008-9471-4 (2009).

Contrasting streamflow regimes induced by melting glaciers across the Tien Shan – Pamir – North Karakoram

Yi Luo[1, 2, 3*], Xiaolei Wang[1, 2], Shilong Piao[4, 5*], Lin Sun[1], Philippe Ciais[6], Yiqing Zhang[2], Changkun Ma[7], Rong Gan[2], Chansheng He[8]

1. Institute of Geographic Science and Natural Resources Research, Chinese Academy of Sciences, Beijing 100101, China

2. University of Chinese Academy of Sciences 19A Yuquan Rd, Shijingshan District, Beijing, 100049, China

3. Xinjiang Institute of Ecology and Geography, Chinese Academy of Sciences, Urumqi, 830011, Xinjiang, China

4. Institute of Tibetan Plateau Research, Center for Excellence in Tibetan Earth Science, Chinese Academy of Sciences, Beijing 100085, China.

5. Sino-French Institute for Earth System Science, College of Urban and Environmental Sciences, Peking University, Beijing 100871, China

6. Laboratoire des Sciences du Climat et de l'Environnement (LSCE), CEA CNRS UVSQ, 91191 Gif Sur Yvette, France.

7. College of Natural Resources and Environment, Northwest A&F University, No.3 Taicheng Road, Yangling 712100, Shaanxi, China

8. Department of Geography, Western Michigan University, 1903 W Michigan Ave Kalamazoo MI 49008-5424, USA

* Corresponding to: Yi Luo, luoyi@igsnrr.ac.cn; Shilong Piao, slpiao@pku.edu.cn

Supplementary Information - Figs

Fig. List

Fig. S1: Fractions of the runoff components based on averages over 1966–1995: glacier runoff, snowmelt runoff and rainfall-runoff (top); ice melt runoff, snowmelt runoff and rainfall-runoff (bottom) for main rivers in the Tien Shan – Pamir – Karakoram. Glacier melt includes supraglacial snowmelt and the runoff of rainfall-over-ice. See Fig. 1 for labelling of river catchments.. 3

Fig. S2: Projected glacier changes in area and water storage in river basins in the Tien Shan – Pamir – Karakoram. See Fig. 1 for labelling of river catchments. 4

Fig. S3: Long-term patterns of change in glacier melt and its components in the main rivers in the Tien Shan-Pamir-Karakoram. See Fig. 1 for labelling of river catchments. 6

Fig. S4 Changes in water balance components of streamflow in the main rivers in the Tien Shan-Pamir-Karakoram. See Fig. 1 for labelling of river catchments. 9

Fig. S5: Projected changes in hydrographs of streamflow and its components for main rivers in the Tien Shan – Pamir – Karakoram under climate change scenarios with comparison to their historical ones. See Fig. 1 for labelling of river catchments.. 12

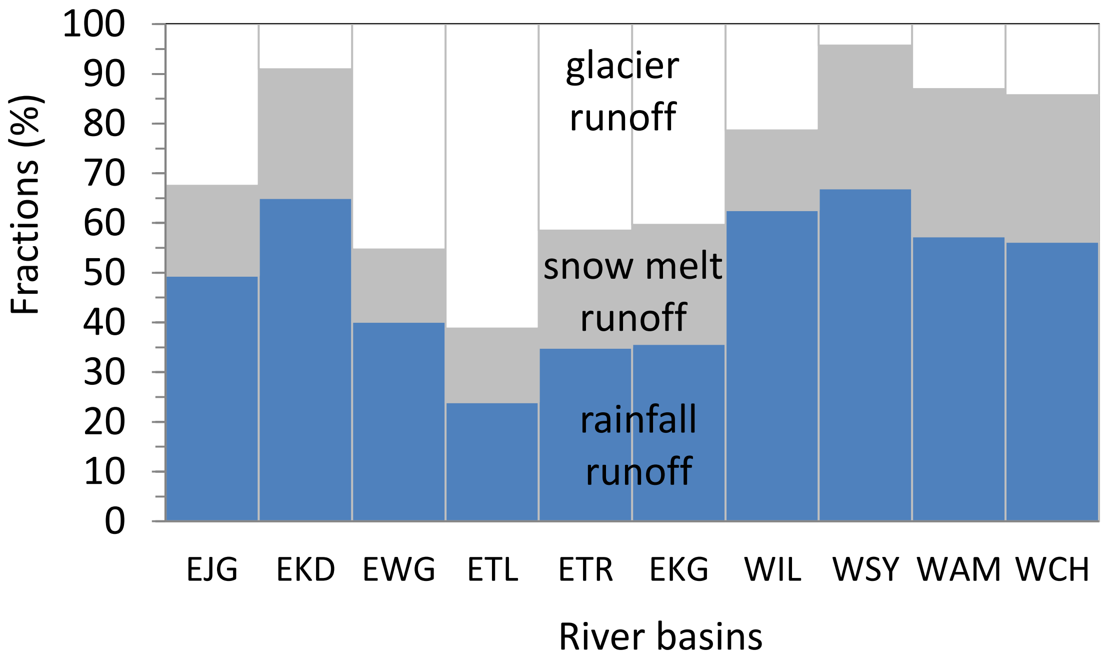

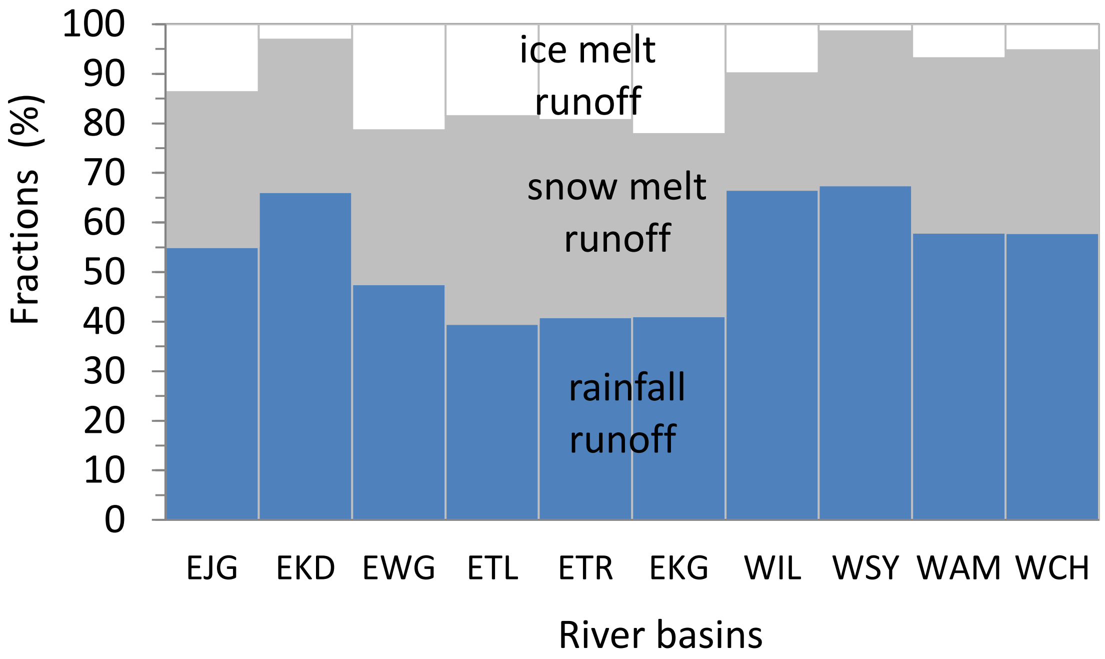

Fig. S1: Fractions of the runoff components based on averages over 1966–1995: glacier runoff, snowmelt runoff and rainfall-runoff (top); ice melt runoff, snowmelt runoff and rainfall-runoff (bottom) for main rivers in the Tien Shan – Pamir – Karakoram. Glacier melt includes supraglacial snowmelt and the runoff of rainfall-over-ice. See Fig. 1 for labelling of river catchments.

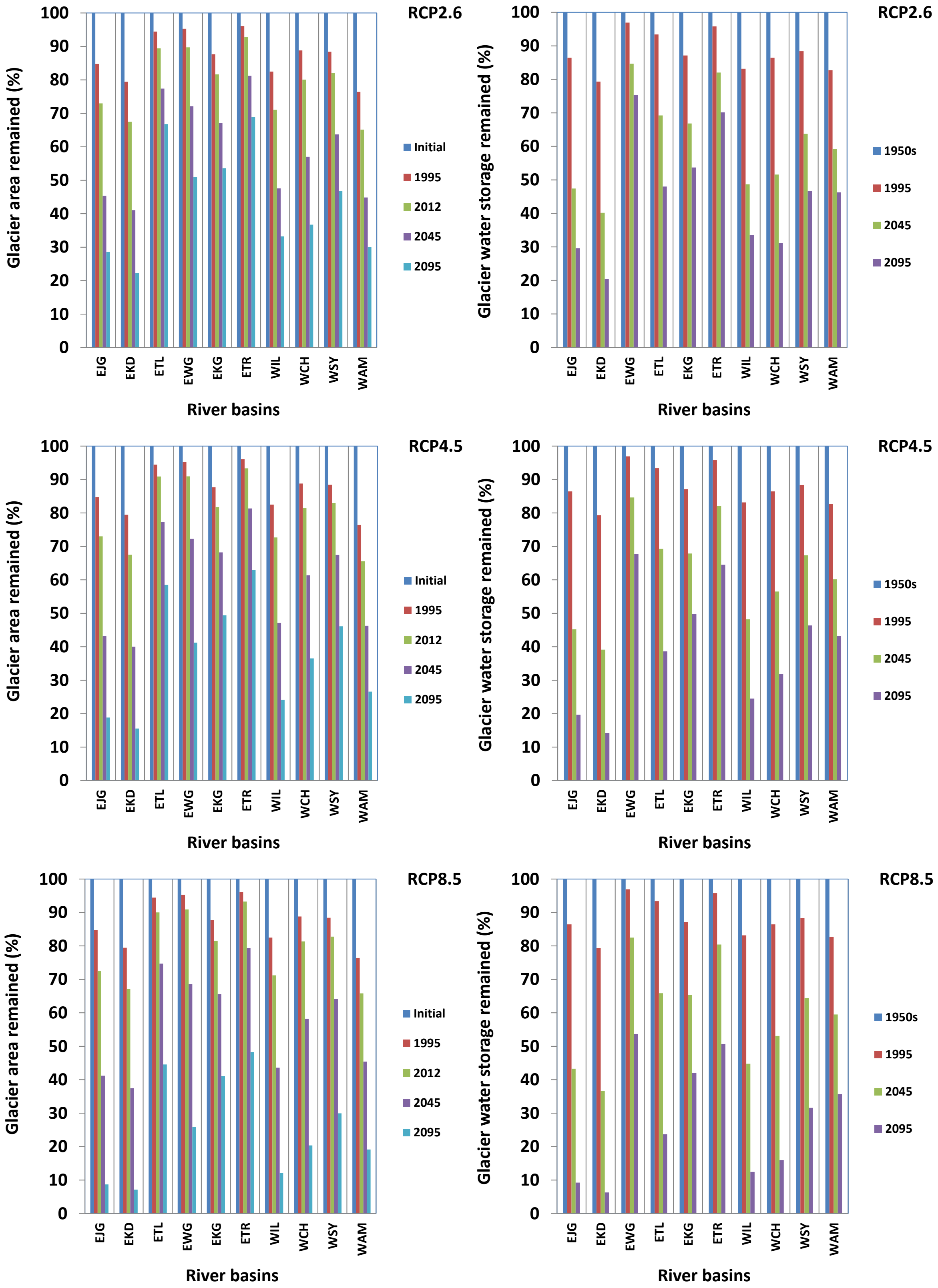

Fig. S2: Projected glacier changes in area and water storage in river basins in the Tien Shan – Pamir – Karakoram. See Fig. 1 for labelling of river catchments.

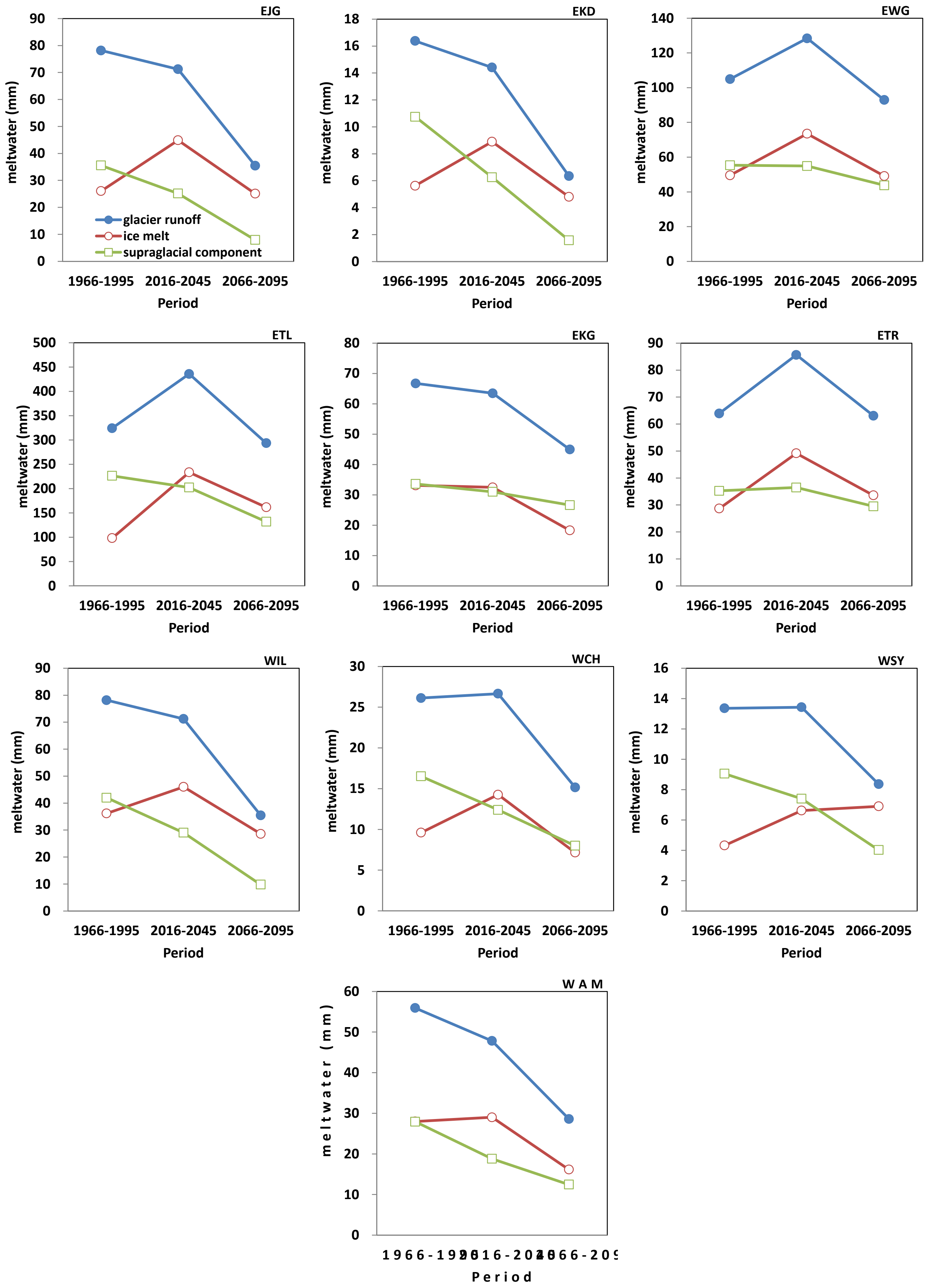
EJG
EKD
EWG
ETL
EKG
ETR
WIL
WCH
WSY
W A M
glacier runoff
ice melt
supraglacial component
meltwater (mm)
1966-1995
2016-2045
2066-2095
Period

(a) RCP2.6

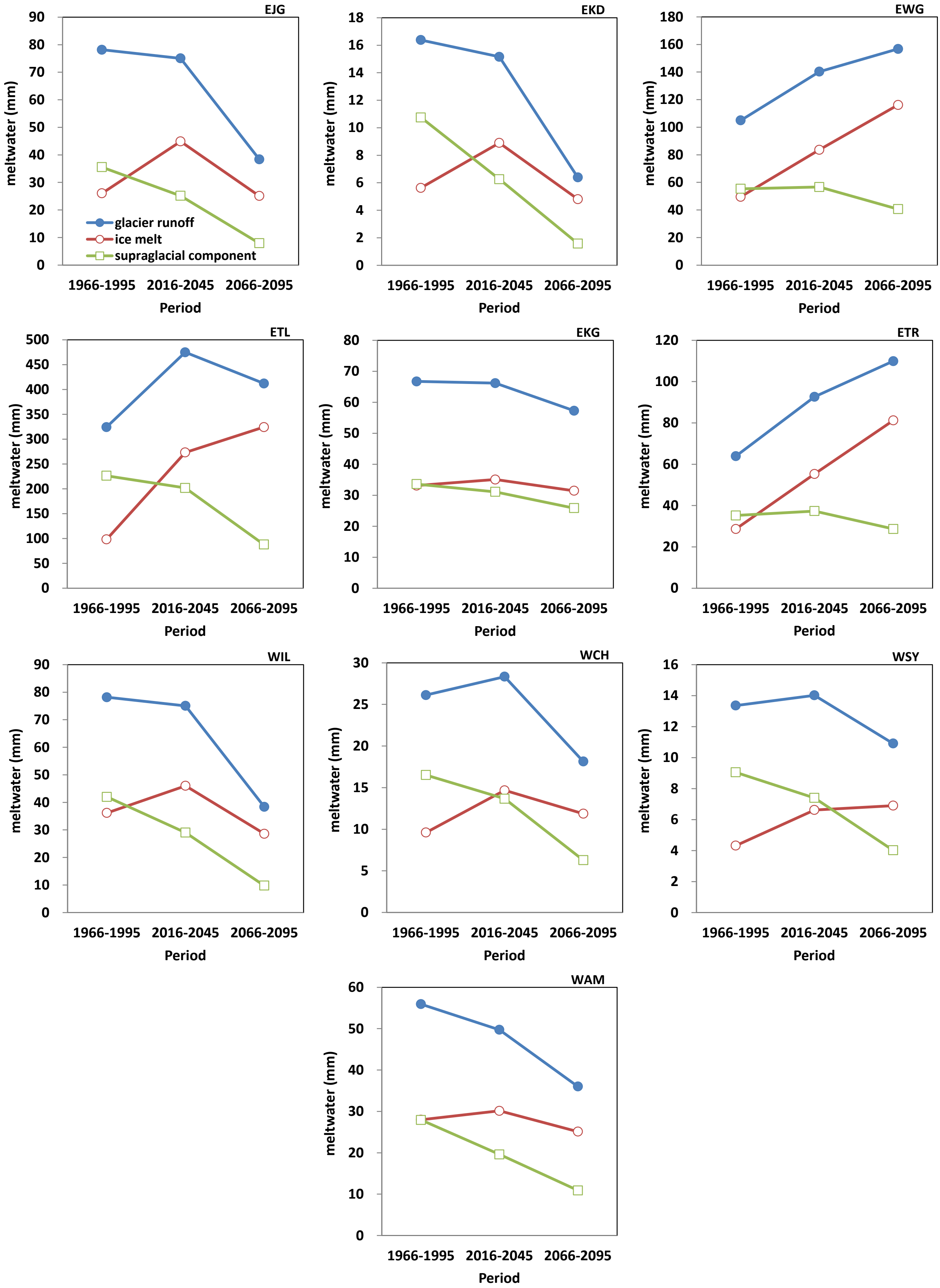

(b) RCP8.5

Fig. S3: Long-term patterns of change in glacier melt and its components in the main rivers in the Tien Shan-Pamir-Karakoram. See Fig. 1 for labelling of river catchments.

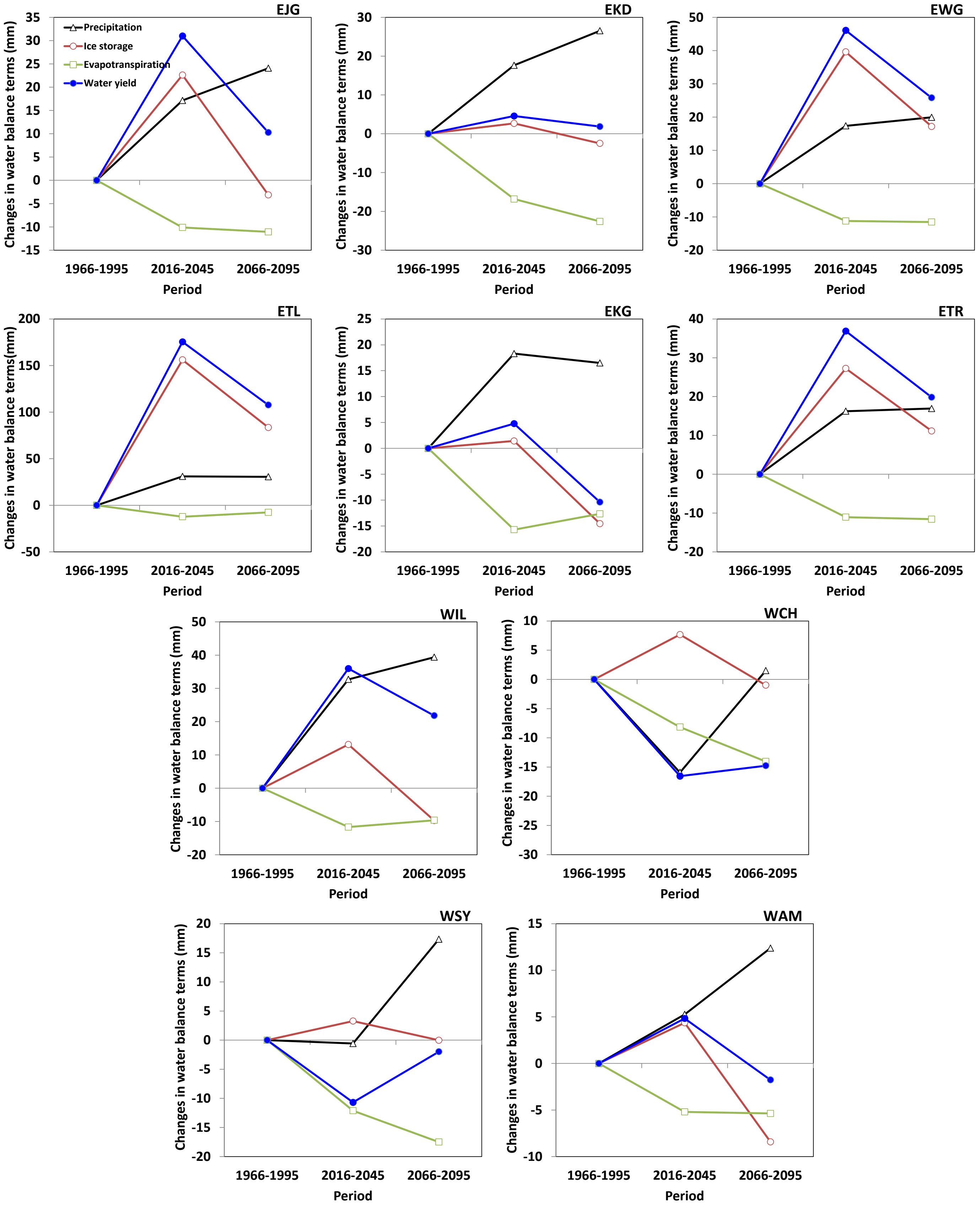
EJG
EKD
EWG
ETL
EKG
ETR
WIL
WCH
WSY
WAM
Precipitation
Ice storage
Evapotranspiration
Water yield
Changes in water balance terms (mm)
1966-1995
2016-2045
2066-2095
Period

(a) RCP2.6

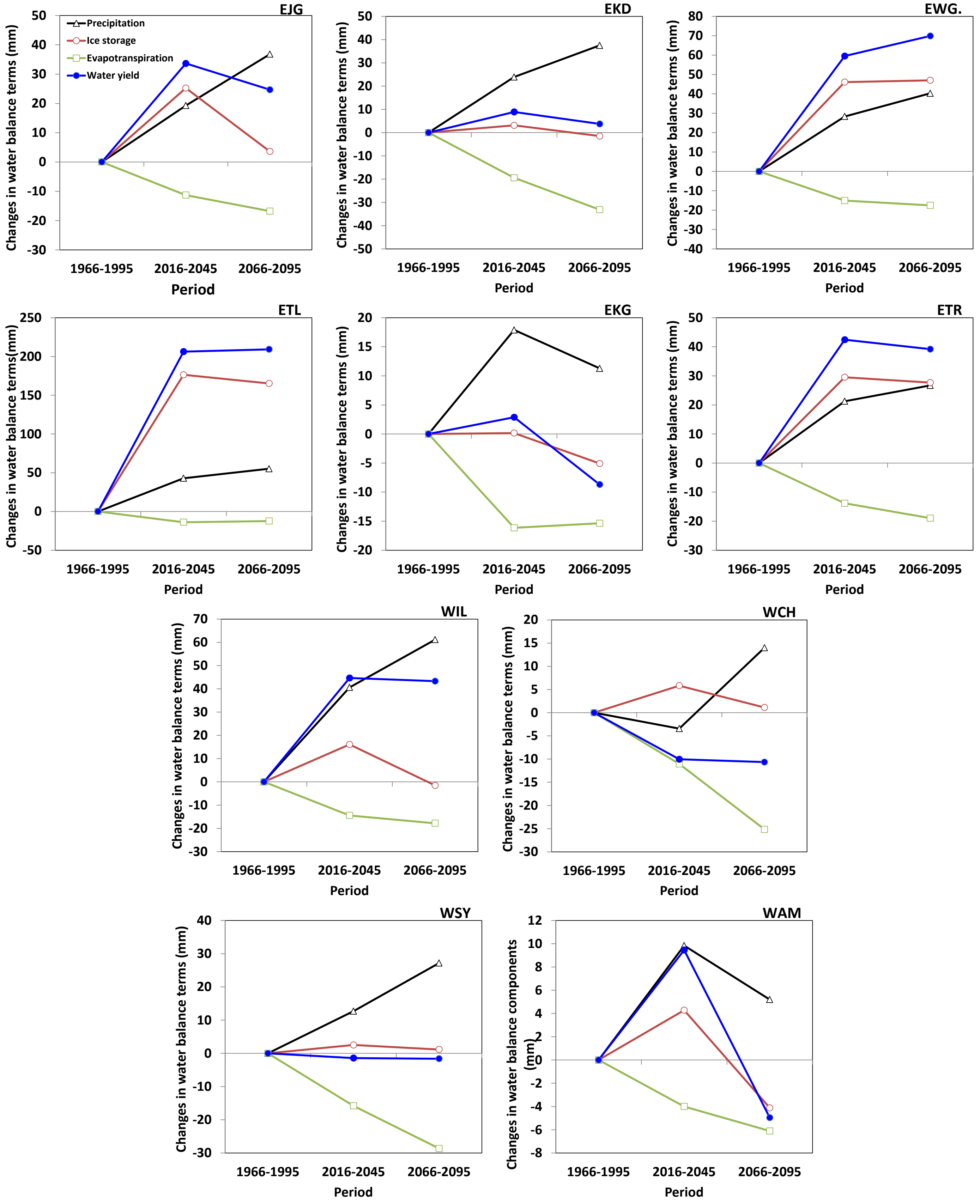
EJG
Precipitation
Ice storage
Evapotranspiration
Water yield
Changes in water balance terms (mm)
1966-1995
2016-2045
2066-2095
Period
EKD
EWG.
ETL
Changes in water balance terms(mm)
EKG
ETR
WIL
WCH
WSY
WAM
Changes in water balance components (mm)

(b) RCP4.5

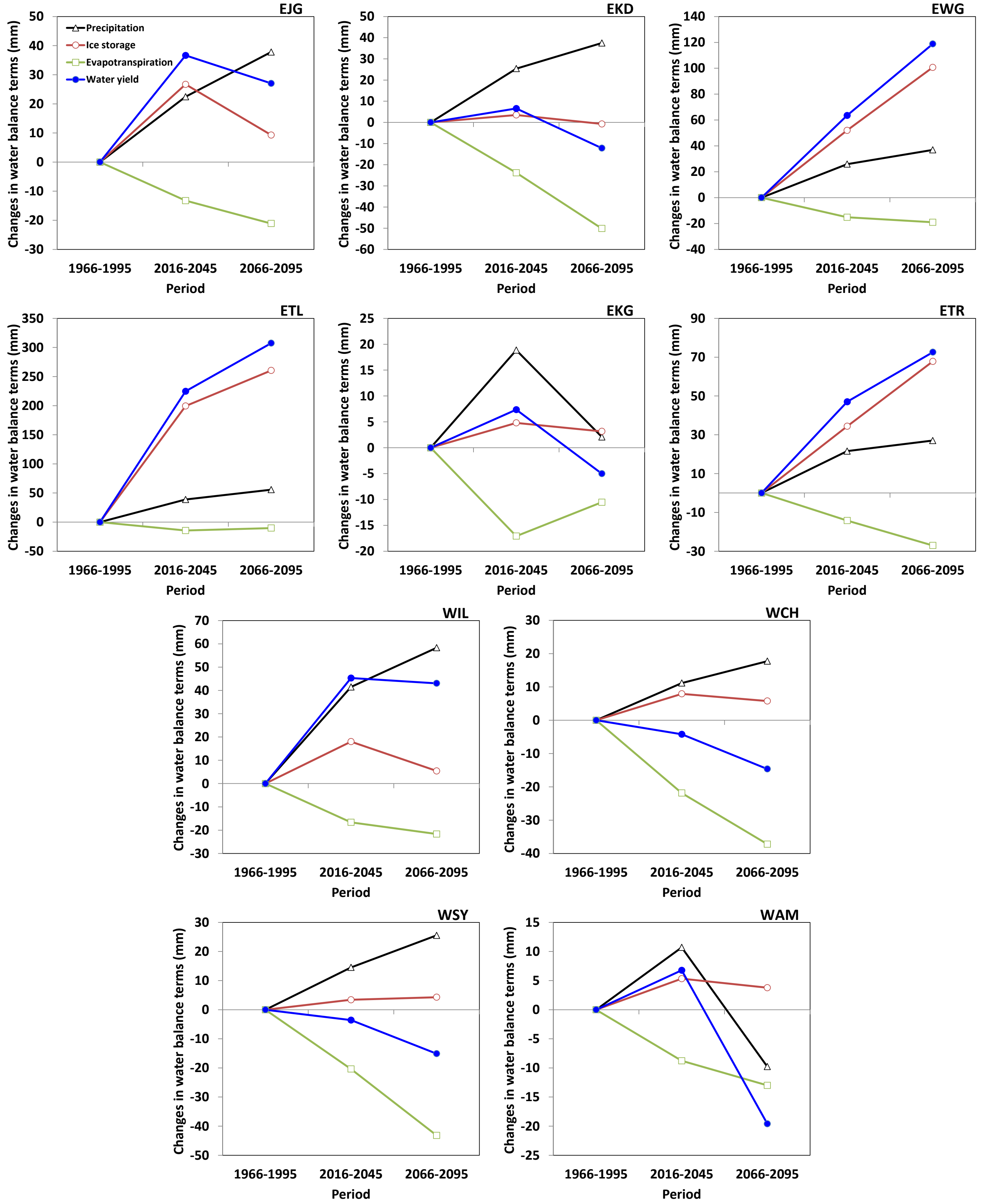

(b) RCP8.5

Fig. S4 Changes in water balance components of streamflow in the main rivers in the Tien Shan-Pamir-Karakoram. See Fig. 1 for labelling of river catchments.

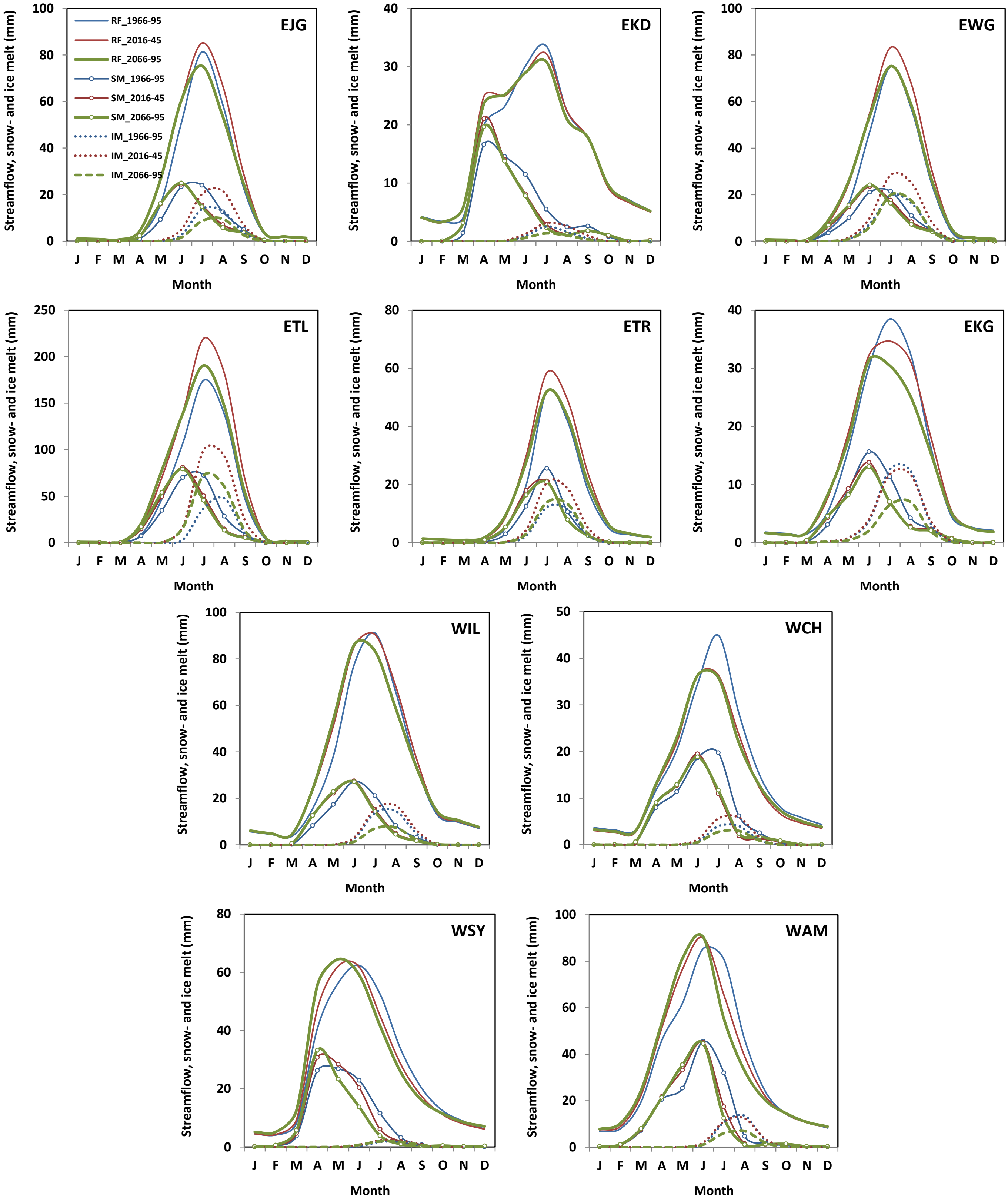
RF_1966-95
RF_2016-45
RF_2066-95
SM_1966-95
SM_2016-45
SM_2066-95
IM_1966-95
IM_2016-45
IM_2066-95
EJG
EKD
EWG
ETL
ETR
EKG
WIL
WCH
WSY
WAM
Streamflow, snow- and ice melt (mm)
Month
J F M A M J J A S O N D

(a) RCP2.6

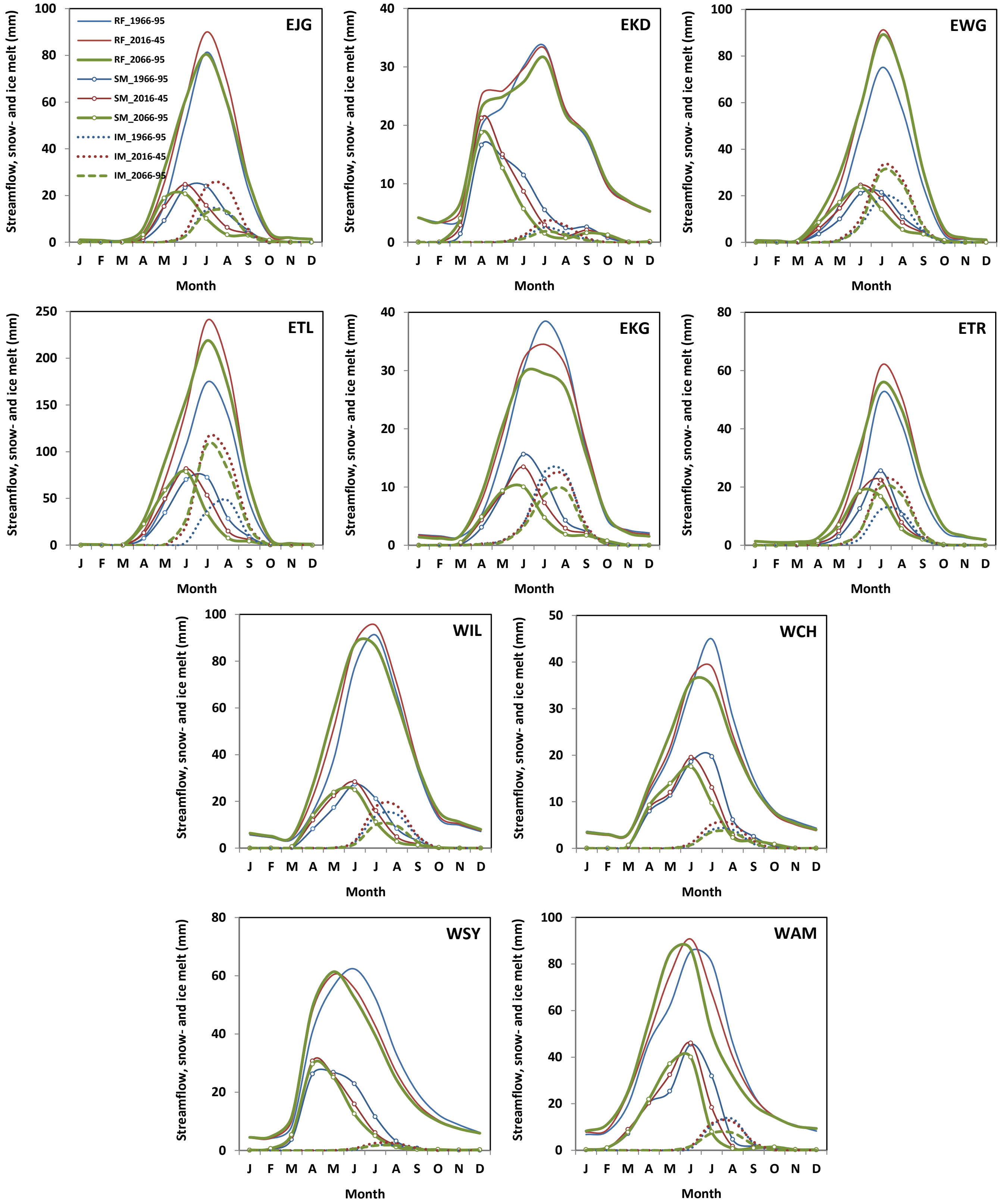
RF_1966-95
RF_2016-45
RF_2066-95
SM_1966-95
SM_2016-45
SM_2066-95
IM_1966-95
IM_2016-45
IM_2066-95
EJG
EKD
EWG
ETL
EKG
ETR
WIL
WCH
WSY
WAM
Streamflow, snow- and ice melt (mm)
J F M A M J J A S O N D
Month

(b) RCP4.5

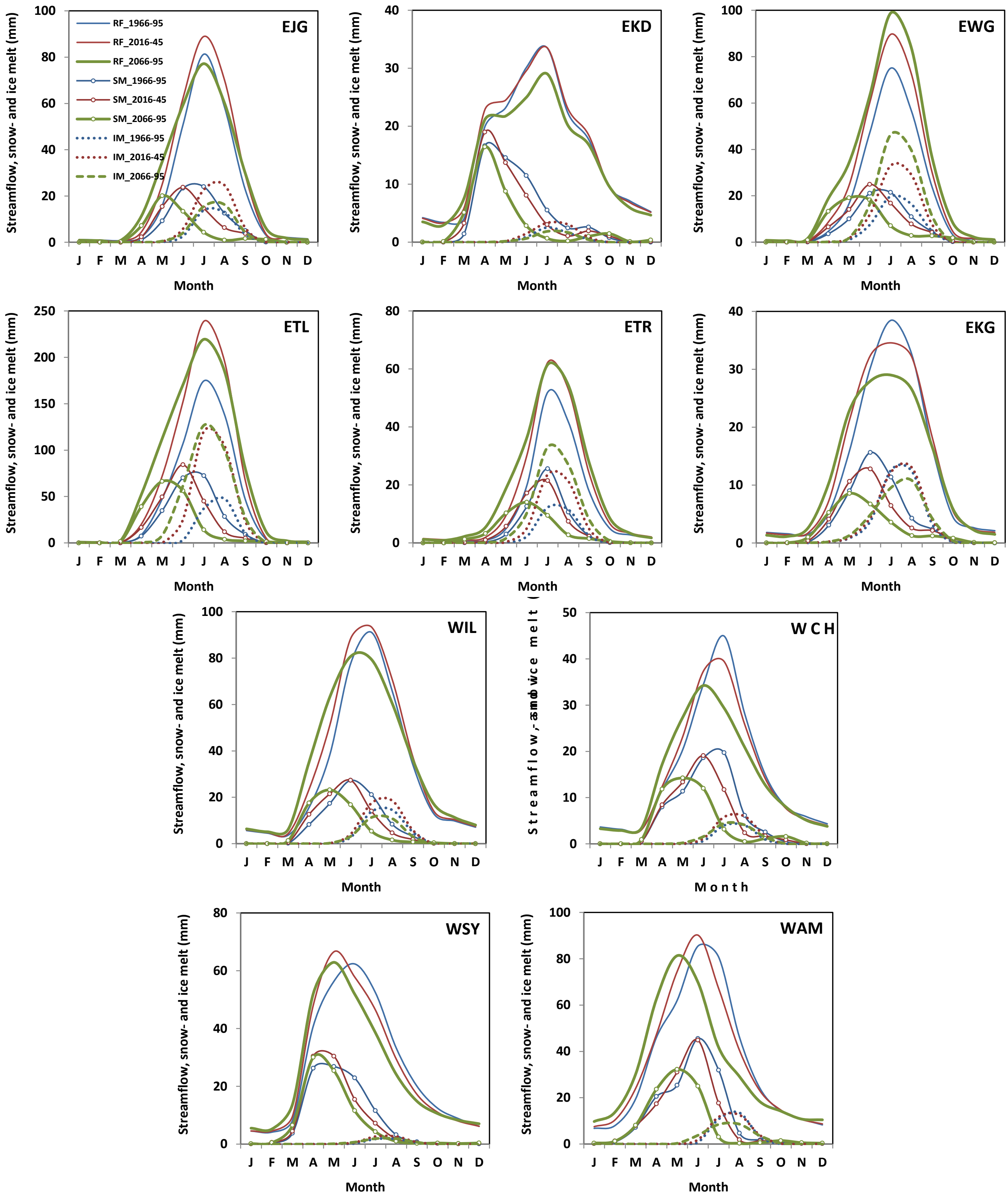

(b) RCP8.5

Fig. S5: Projected changes in hydrographs of streamflow and its components for main rivers in the Tien Shan – Pamir – Karakoram under climate change scenarios with comparison to their historical ones. See Fig. 1 for labelling of river catchments.

Contrasting streamflow regimes induced by melting glaciers across the Tien Shan – Pamir – North Karakoram

Yi Luo[1, 2, 3*], Xiaolei Wang[1, 2], Shilong Piao[4, 5*], Lin Sun[1], Philippe Ciais[6], Yiqing Zhang[2], Changkun Ma[7], Rong Gan[2], Chansheng He[8]

1. Institute of Geographic Science and Natural Resources Research, Chinese Academy of Sciences, Beijing 100101, China

2. University of Chinese Academy of Sciences 19A Yuquan Rd, Shijingshan District, Beijing, 100049, China

3. Xinjiang Institute of Ecology and Geography, Chinese Academy of Sciences, Urumqi, 830011, Xinjiang, China

4. Institute of Tibetan Plateau Research, Center for Excellence in Tibetan Earth Science, Chinese Academy of Sciences, Beijing 100085, China.

5. Sino-French Institute for Earth System Science, College of Urban and Environmental Sciences, Peking University, Beijing 100871, China

6. Laboratoire des Sciences du Climat et de l'Environnement (LSCE), CEA CNRS UVSQ, 91191 Gif Sur Yvette, France.

7. College of Natural Resources and Environment, Northwest A&F University, No.3 Taicheng Road, Yangling 712100, Shaanxi, China

8. Department of Geography, Western Michigan University, 1903 W Michigan Ave Kalamazoo MI 49008-5424, USA

* Corresponding to: Yi Luo, luoyi@igsnrr.ac.cn; Shilong Piao, slpiao@pku.edu.cn

Supplementary Information - Tables

Table list

Table S1: Physical attributes of source regions for the ten main river basins in Tien Shan-Pamir-Karakoram. See Fig. 1 for labelling of river catchments..3

Table S2: Estimation of the components and their proportions in the streamflow of the main river basins in Tien Shan – Pamir - Karakoram, based on simulations and averaged over 1966-1995. See Fig. 1 for labelling of river catchments..5

Table S3: Projected glacier area and ice-water storage in 2045 and 2095 in the source regions of the ten main river basins in Tien Shan – Pamir – Karakoram. See Fig. 1 for labelling of river catchments. 6

Table S4: Ice-melt in different periods in the source regions of the ten main river basins in Tien Shan – Pamir – Karakoram. See Fig. 1 for labelling of river catchments. 7

Table S1: Physical attributes of source regions for the ten main river basins in Tien Shan-Pamir-Karakoram. See Fig. 1 for labelling of river catchments.

Sub-regions	Rivers	Tributaries		Upper stream basins			Elevations			Glaciers				
				Area	sub-basins	HRUs and GHRUs	Bottom	Top	Mean	Area	No.	RGA		Inventories
				km^2			m a.s.l.			km^2		%		Year
Eastern Tien Shan	EJG	Baiyang		271	5	53	1020	5377	2762	21	15	7.8	8.9	1962
		Urumqi		923	9	192	1883	4450	3121	38	124	4.1		1964
		Hutubi		1809	19	389	1252	5093	3022	72	239	4.0		
		Manas		5163	50	1149	846	5145	3252	608	800	11.8		
		Sikeshu		914	5	181	1067	4521	3057	106	128	11.6		1963/1964
		Jing		1409	7	167	685	4340	2635	91	116	6.5		1959
	WIL	Kashi		9551	100	1214	798	4615	2309	422	551	4.4	7.2	1962/1964/1966
Central Tien Shan		Kukesu		5638	57	990	1211	4795	2990	423	627	7.5		1962/1963/1974
		Aheyazi		2636	25	610	1737	5963	3441	549	440	20.8		1963/1976
		Tekes		9112	87	829	1638	5690	2590	556	378	6.1		1962/1963/1973/1974/1976
	EKD			18650	186	1920	1097	4796	2900	423	689	2.3	2.3	1962/1963/1964
	EWG	Kuqa		2912	31	212	1271	4488	2575	24	59	0.8	13.5	1963
		Hez		3541	33	237	1312	4802	2482	43	78	1.2		
		Kalasu		1309	9	133	1385	5026	2721	66	76	5.0		1962/1963
		Kamusilang		1843	13	246	1491	5995	3268	299	157	16.2		
		Muzat		2659	19	363	1869	6779	3959	1219	254	45.9		1962/1963/1964/1971
	ETL			1364	11	159	1568	7126	3685	444	105	32.6	32.6	1976
	ETR	Aksu	Kumaric	12990	99	1577	1434	7070	3732	2878	1269	22.2	12.4	1973/1976/1980
			Toshgan	19470	206	1461	1888	5934	3565	811	677	4.2		1973/1980
East Pamir and Karakoram		Yarkant		46746	414	6059	1446	8515	4423	5528	2682	11.8		1963/1968/1971/1976/1977
		Hortan	Eron Kash	14813	129	2291	1275	6841	4694	2954	1334	19.9		1968/1970/1971
			Karakax	21532	181	3448	1566	6720	4714	2170	2010	10.1		1968/1976/1980/1986
	EKG		Kezi	14180	131	1452	1710	6576	3614	897	527	6.3	9.5	1946/1956/1959/1960/1963
			Gez	9204	90	1261	2182	7539	4339	1216	486	13.2		1959/1960

													/1963/1964
		Kushan	2191	18	321	1996	7577	4007	316	123	14.4		1963/1964
Western Tien Shan	WCH		9548	67	610	1249	4617	2933	241	315	2.5	2.5	1950s
	WSY	Naryn	58205	551	3864	388	5118	2737	1186	1528	2.0	2.2	
		Kara	20351	271	1666	398	4977	2062	294	512	1.4		
		Chirchik	11725	235	1553	248	4376	2296	184	690	1.6		
		Ferghana	11118	112	1274	742	5529	2607	590	352	5.3		
Pamir	WAM	Panj	106225	886	12081	302	7324	3716	6199	6240	5.8	6.6	1950s
		Vakhsh	32380	325	3719	300	7441	3805	3909	2207	12.1		
		Kafirnigan	6035	96	676	306	4801	2673	107	268	1.8		
		Surkhan	6170	153	813	290	4737	2572	94	250	1.5		
		Zeravshan	10561	140	1291	899	5478	3192	664	751	6.3		
		Kashka	5846	161	673	293	4382	2500	18	44	0.3		

Table S2: Estimation of the components and their proportions in the streamflow of the main river basins in Tien Shan – Pamir - Karakoram, based on simulations and averaged over 1966-1995. See Fig. 1 for labelling of river catchments.

Runoff and components		Western slope rivers				Eastern slope rivers					
		WIL	WCH	WSY	WAM	EJG	EKD	EWG	ETL	EKG	ETR
Water yield, Q	mm	366	183	310	412	241	180	232	530	150	148
Glacier runoff, Q_g		78	26	13	56	78	16	105	324	67	64
Ice melt, Q_i		36	10	4	28	33	6	50	98	33	29
Supraglacial snowmelt, Q_{gs}		27	13	7	23	32	9	38	143	19	24
Non-glacial snowmelt, Q_{ngs}		59	53	89	116	44	47	34	79	27	31
Snowmelt, Q_s		86	66	96	139	75	55	72	222	46	55
Q_g / Q	%	21	14	4	13	33	9	45	61	40	41
Q_i / Q		10	5	1	7	14	3	21	19	22	19

Table S3: Projected glacier area and ice-water storage in 2045 and 2095 in the source regions of the ten main river basins in Tien Shan – Pamir – Karakoram. See Fig. 1 for labelling of river catchments.

River basins	Glacier-water storage				Glacier area			
	1950-60s	1995	2045	2095	1950-60s	1995	2045	2095
	km^3	Relative to 1950-60s (%)			km^2	Relative to 1950-60s (%)		
	RCP2.6							
EJG	52	86	47	30	936	85	45	29
EKD	21	79	40	20	423	79	41	22
EWG	254	97	85	75	1650	94	77	67
ETL	62	93	69	48	444	95	72	51
EKG	223	87	67	54	2429	88	67	54
ETR	1747	96	82	70	14341	96	81	69
WIL	142	83	49	34	1949	82	48	33
WCH	20	86	52	31	241	89	57	37
WSY	143	88	64	47	2254	88	64	47
WAM	972	83	59	46	10991	76	45	30
Eastern slope rivers + WIL	2500	94	78	65	22172	93	74	61
WCH + WSY + WAM	1133	83	60	46	13465	78	48	32
	RCP4.5							
EJG	52	86	45	20	936	85	43	19
EKD	21	79	39	14	423	79	40	16
EWG	254	97	85	68	1650	94	77	58
ETL	62	93	69	39	444	95	72	41
EKG	223	87	68	50	2429	88	68	49
ETR	1747	96	82	65	14341	96	81	63
WIL	142	83	48	25	1949	82	47	24
WCH	20	86	56	32	241	89	61	37
WSY	143	88	67	46	2254	88	67	46
WAM	972	83	60	43	10991	76	46	27
Eastern slope rivers + WIL	2500	94	78	59	22172	93	74	55
WCH + WSY + WAM	1133	83	60	43	13465	78	50	30
	RCP8.5							
EJG	52	86	43	9	936	85	41	9
EKD	21	79	37	6	423	79	37	7
EWG	254	97	82	54	1650	94	75	45
ETL	62	93	66	24	444	95	69	26
EKG	223	87	65	42	2429	88	66	41
ETR	1747	96	80	51	14341	96	79	48
WIL	142	83	45	12	1949	82	44	12
WCH	20	86	53	16	241	89	58	20
WSY	143	88	64	32	2254	88	64	30
WAM	972	83	60	36	10991	76	45	19
Eastern slope rivers + WIL	2500	94	76	46	22172	93	72	41
WCH + WSY + WAM	1133	83	60	35	13465	78	48	21

Table S4: Ice-melt in different periods in the source regions of the ten main river basins in Tien Shan – Pamir – Karakoram. See Fig. 1 for labelling of river catchments.

	Ice-melt (mm)						
	Reference	RCP2.6		RCP4.5		RCP8.5	
River basins	1966-1995	2016-2045	2066-2095	2016-2045	2066-2095	2016-2045	2066-2095
EJG	26	41	16	43	21	45	25
EKD	6	8	3	9	4	9	5
EWG	50	73	49	79	72	84	116
ETL	98	234	162	253	236	273	324
EKG	32	18	33	25	33	31	28
ETR	29	49	34	52	47	55	81
WIL	36	42	18	45	24	46	29
WCH	10	14	7	13	9	15	12
WSY	4	6	4	6	5	7	7
WAM	28	29	16	30	19	30	25
